# Leaf seasonal osmotic adjustment is not driven by temperature or water deficit

**DOI:** 10.1111/tpj.70569

**Published:** 2025-11-14

**Authors:** Elena Farolfi, Adéla Kulhánková, Federica De Berardinis, Soma László Tarnay, Gregory A. Gambetta, Uri Hochberg, Astrid Forneck, Jose Carlos Herrera

**Affiliations:** ^1^ Institute of Viticulture and Pomology, Department of Agricultural Sciences BOKU University Tulln an der Donau Austria; ^2^ Department of Horticulture, Faculty of Agrobiology, Food and Natural Resources Czech University of Life Sciences Prague Prague Czech Republic; ^3^ EGFV, Bordeaux Sciences Agro, INRAE Université de Bordeaux, ISVV Villenave‐d'Ornon France; ^4^ Institute of Soil, Water and Environmental Sciences Volcani Center, ARO Ramat Yishai Israel

**Keywords:** grapevine, osmolytes, osmotic adjustment, osmotic potential, temperature, water deficit

## Abstract

Osmotic adjustment (OA), the active accumulation of osmolytes in plant cells, plays a crucial role in plant‐drought response. OA occurs throughout the growing season, but the seasonal effects of temperature and water status on OA remain unclear. We investigated grapevine (*Vitis vinifera*) leaves' regulation of osmotic potential over time, across different temperatures and water deficit conditions and the contributions of sugars and cations driving these changes. Potted grapevines were grown under greenhouse‐controlled conditions at 20, 25 and 30°C, with half of the vines being exposed to mild water deficit (ca. −1 MPa) for 35 days. Leaf osmotic potential at full turgor (π_100_) was measured via osmometer, while sugar and cation concentrations were quantified by HPIC. Furthermore, π_100_ was also monitored in field‐grown vines. Our findings confirmed seasonal OA in grapevine, with a progressive decrease in π_100_ due to solute accumulation. However, the adjustment rate was not significantly influenced by temperature or mild drought, although both factors affected absolute π_100_. Sugars and cations accounted for 54.5% of the π_100_, with little variation over the season, suggesting OA is not driven by a single osmolyte. These findings support the idea that plants from Mediterranean/temperate climates follow a pre‐programmed seasonal OA strategy, largely independent of environmental variability.

## Significance Statement

The study provides evidence of a seasonal osmotic adjustment in grapevine (*Vitis vinifera* cv. Pinot noir). The rate of seasonal osmotic adjustment is mostly independent of the ambient temperature conditions and the plant water status.

## INTRODUCTION

Plant cell hydration relies on two physical structures: the plasma membrane, a semipermeable membrane that selectively regulates the movement of water and solutes, and the cell wall, which provides structural support as water enters the cell by resisting overexpansion, ensuring that the cell remains firm (Ali et al., 2023). Water influx, driven by the osmotic gradient between the symplast and the external environment, generates turgor pressure, the positive hydrostatic pressure exerted by the protoplast against the cell wall (Beauzamy et al., 2014). The osmotic potential (π) of the cell, determined by its solute concentration, is always negative and defines the tendency of water to move due to solute differences. It also determines the direction of water movement via osmosis (Hsiao et al., 1976; Morgan, 1984). A turgid cell results from the balance between turgor pressure and osmotic potential, which is essential for plant structure, growth and vital physiological processes (Ali et al., 2023; Hsiao et al., 1976; Zimmermann, 1978). Turgor loss due to environmental stressors, such as drought or salinity, threatens the physiological processes of the cells, leading to reduced growth, plant hydraulic dysfunction and plasmolysis (Morgan, 1984; Seelig et al., 2015; Turner, 1981). To maintain turgor under challenging conditions, plants actively accumulate or synthesize a wide range of osmotically active compounds, defined as osmolytes, such as sugars, amino acids and ions, with some variability among species (Chaves & Oliveira, 2004; Hellebust, 1976; Wang & Stutte, 1992). This process, known as osmotic adjustment, enables plants to sustain turgor pressure even under conditions of reduced water potential caused by environmental stresses such as drought or high salinity (Abrams, 1988c; Morgan, 1984; Patakas & Nortsakis, 1999).

Osmotic adjustment (OA) is widely recognised as a key drought resistance trait in many plant species, as it helps maintain stomatal conductance even under low water potential conditions, thereby supporting essential physiological functions (Bartlett et al., 2012; Blum, 2017). Understanding how this process enhances drought resilience is especially important in the context of climate change, where rising temperatures and shifting precipitation patterns increasingly threaten cell turgor maintenance. Other than drought, shifts towards more negative osmotic potential at full turgor (π_100_) have been observed to occur throughout the vegetative season in various plants from Mediterranean/temperate climates (Abrams, 1988a; Alsina et al., 2007; Bahari et al., 1985; Gersony et al., 2020; Herrera et al., 2022; Hinckley et al., 1980; O'Neill, 1983; Sinclair et al., 2024; Sorek et al., 2021, 2022). However, it is not clear if such seasonal osmotic adjustment is triggered in response to progressive environmental hardening as rising temperatures and decreasing water availability become more prevalent (Herrera et al., 2022; Sorek et al., 2021). Indeed, the interactive effect of environmental variables such as temperature and water status on OA remains largely unknown (Herrera & Hochberg, 2024).

Due to its global cultivation and economic importance, grapevine physiology has been extensively studied, making it a valuable model plant for investigating physiological mechanisms such as OA under various stress conditions (Gambetta et al., 2020). Previous studies on grapevine that focused on the OA in response to drought identified soluble sugars (Patakas et al., 2002; Perry et al., 2022; Rodrigues et al., 1993) and cations (Degu et al., 2019) as the primary osmolytes. While other compounds such as proline are involved in stress response, their contribution to the osmotic potential has been reported to be minimal in grapevines under drought (Patakas & Nortsakis, 1999).

This study aimed to shed light on the combined effects of temperature and water deficits on seasonal changes in π_100_, using grapevine as a model plant. Specifically, we examined whether seasonal solute accumulation, which leads to lower π_100_, is driven by temperature variations and/or water availability throughout the growing season. To disentangle the effects of temperature and water deficit on leaf seasonal osmotic adjustment, we conducted complementary greenhouse and field studies. In the greenhouse, plants were grown under three constant temperature regimes and subjected to controlled water deficits. In the field, we monitored π_100_ in fully irrigated grapevines exposed to natural field temperature variations. Finally, we assessed the relative contributions of soluble sugars and cations to the OA.

## RESULTS

### Seasonal variations in leaf osmotic potential

**Figure 1:**
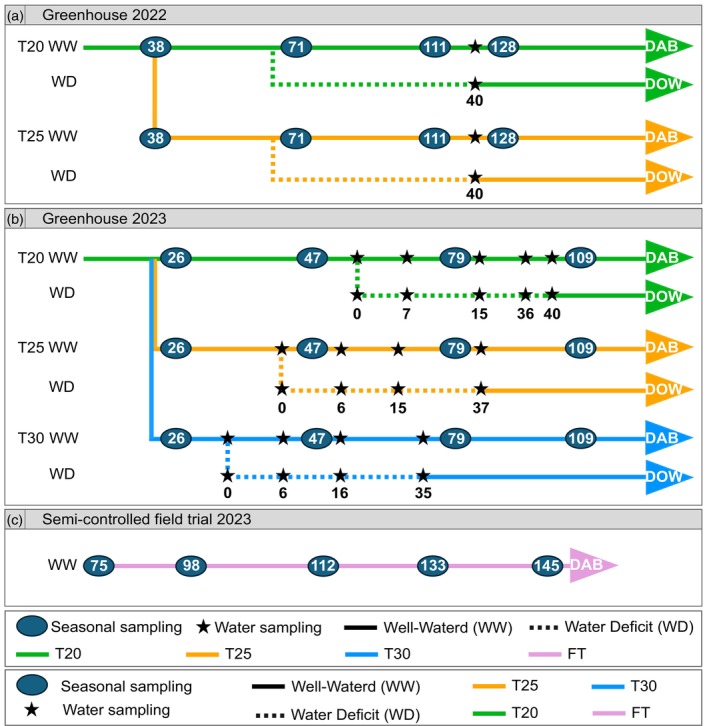
Experimental design to assess variation in grapevine leaf osmotic potential at full turgor (π_100_) in greenhouse season 2022 (a), season 2023 (b) and the semi‐controlled field (c). Solid lines represent the well‐watered period, while dotted lines indicate the water deficit period. Different temperatures are represented by the following line colours: green represents 20°C/15°C day/night (T20), orange 25°C/20°C day/night (T25), blue 30°C/25°C day/night (T30), and lilac denotes uncontrolled temperatures from the semi‐controlled trial (FT). Full circles represent sampling dates aimed at testing seasonal osmotic adjustment under well‐watered conditions, based on days after budbreak (DAB). Stars mark sampling dates intended to evaluate the response of π_100_ to water deficit, with time references at the onset of water deficit (DOW).

Our study aimed to investigate changes in the leaf osmotic potential at full turgor (π_100_) over the season, focusing on the potential variation induced by temperature. To test the effect of temperature, leaves from well‐watered plants were collected at consecutive sampling times for osmometer measurements (Figure 1). The samples were collected from greenhouse chambers, where plants were growing in specific temperature conditions, and from semi‐controlled field trial to track the seasonality.

**Figure 2:**
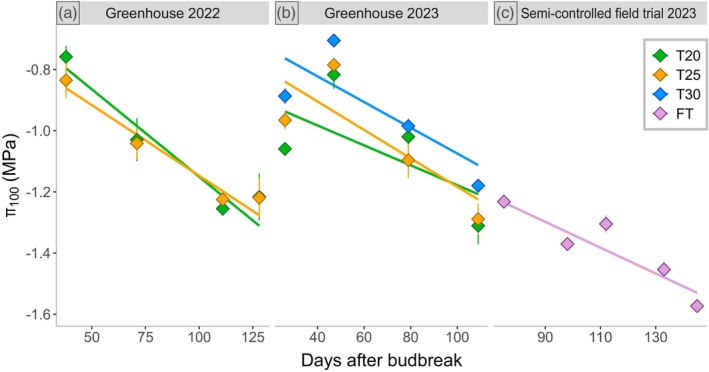
Seasonal variation of leaf osmotic potential at full turgor (π_100_) over days after budbreak in grapevine leaves from the greenhouse season 2022 (a), season 2023 (b) and the semi‐controlled field (c) for well‐watered plants. Different colours represent various growth temperatures for day/night: green represents 20°C/15° (T20), orange 25°C/20°C (T25), blue 30°C/25°C (T30), and lilac denotes the environmental temperature of the semi‐controlled field trial (FT). Each point represents the mean of π_100_ ± SE; (semi‐controlled field trial *n* = 11–22; greenhouse 2022 *n* = 3–6; greenhouse 2023 *n* = 4–9). Solid lines indicate the linear regression of all data points within the respective temperature. ANOVA reported π_100_ is statistically impacted by DAB (*P* < 0.001) and by T30 (*P* < 0.01). Details on statistical analysis are provided in Tables S3–S5.

In the grapevines growing in greenhouse chambers, leaf π_100_ exhibited a consistent shift towards more negative values throughout the season (*P* < 0.001) in the two consecutive years of trials (Figure 2a,b; Tables 1 and 2; Tables S4 and S5). In detail, plants growing at day temperatures of 20°C (T20) and 25°C (T25) in both years exhibited similar π_100_ values (*P* = 0.74; *P* = 0.42), shifting π_100_ to more negative values from −0.79 ± 0.03 MPa at 38 day after budbreak (DAB) to −1.22 ± 0.04 MPa at 128 DAB in season 2022, and from −1.01 ± 0.02 MPa at 26 DAB to −1.30 ± 0.04 MPa at 109 DAB in season 2023.

**Table 1:** Osmotic potential at full turgor (π_100_; MPa) at the end of the water deficit period (day of water deficit 40), seasonal average stem water potential (Ψ_STEM_; MPa) and stomatal conductance (*g*_s_; mol m^−2^ sec^−1^) of plants grown at daytime temperature of 20°C (T20) and 25°C (T25) in the greenhouse during the 2022 season under two different water regimes: well‐watered (WW) and water deficit (WD)

	π_100_	Ψ_STEM_	*g*_s_
Temperature	ns	ns	***
T20	−1.25 ± 0.03	−0.70 ± 0.05	0.11 ± 0.005
T25	−1.24 ± 0.02	−0.68 ± 0.03	0.17 ± 0.009
Water	ns	***	***
WW	−1.23 ± 0.02	−0.52 ± 0.02	0.21 ± 0.007
WD	−1.26 ± 0.04	−0.97 ± 0.04	0.07 ± 0.005
Temperature × Water	ns	ns	***

Values are averages ± SE. *P*‐values were obtained using a two‐way ANOVA. Significances are indicated as follows: ***P < 0.001, ns, *P* > 0.05.

**Table 2:** *P*‐values from the analysis of variance (ANOVA) testing the effects of days after budbreak (DAB), temperature (T), day of water deficit (DOW), water treatment (W) and their interactions on the osmotic potential at full turgor (π_100_) across experiments. Post‐hoc pairwise (Tukey) contrasts between the three greenhouse temperature conditions (T20, T25, T30 for day temperature of 20, 25, 30°C, respectively) were performed for greenhouse 2023

Experiment	Statistical model	Statistical analysis	Effects	*P*‐values
Greenhouse 2022	π_100_ ~ DAB × T	Two‐way ANOVA	DAB	**<0.001**
			T	0.740
			DAB × T	0.313
Greenhouse 2023	π_100_ ~ DAB × T	Two‐way ANOVA	DAB	**<0.001**
			T	**0.004**
			DAB × T	0.580
		Post‐hoc	T20–T25	0.419
		Post‐hoc	T20–T30	**0.003**
		Post‐hoc	T25–T30	0.079
Greenhouse 2023	π_100_ ~ DOW × W × T	Three‐way ANOVA	DOW	**<0.001**
			W	**<0.001**
			T	**<0.001**
			DOW × W	0.497
			DOW × T	0.797
			T × W	0.803
			DOW × W × T	0.723
		Post‐hoc	T20–T25	0.752
		Post‐hoc	T25–T30	**<0.001**
		Post‐hoc	T20–T30	**<0.001**
Semi‐controlled field trial	π_100_ ~ DAB	One‐way ANOVA	DAB	**<0.001**

Significant *P*‐values (*P* < 0.05) are shown in bold.

In season 2023, one further greenhouse chamber with a day temperature of 30°C (T30) was introduced to the experimental setup. Consistent with the previous year, no significant differences in π_100_ were observed between T20 and T25 (*P* = 0.41). However, a notable temperature‐induced effect was detected at T30, as shown in Figure 2c. Plants grown at T30 exhibited significantly less negative π_100_ values compared to T20 (−0.11 MPa, *P* < 0.01) and showed a similar trend when compared to T25 (−0.09 MPa, *P* = 0.08). This pattern persisted throughout the growing season, with π_100_ values ranging from −0.89 ± 0.03 MPa at DAB 26 to −1.18 ± 0.02 MPa at DAB 109 (Table 2; Table S5). However, the progression of the season on π_100_ did not differ significantly between temperature levels (Table 2; Tables S4 and S5) in seasons 2022 (*P* = 0.31) and 2023 (*P* = 0.70), indicating a consistent relationship with time across temperatures.

Similarly to what was observed in greenhouse conditions, the π_100_ of grapevines growing under semi‐controlled field conditions, significantly decreased during the season from −1.23 ± 0.02 MPa at the first sampling date (75 days after budbreak; DAB), to a minimum of −1.57 ± 0.02 MPa 70 days after, at 145 DAB (Figure 2c; Table 2; Table S3).

### Seasonal variations in osmotic potential as affected by drought

To disentangle the effect of water deficit and temperature in the shift of π_100_, a comparable water deficit regime was imposed in the greenhouse chambers during seasons 2022 and 2023, as illustrated in Figure 1(a,b). In the 2022 season, water deficit (WD) plants exhibited significantly lower Ψ_STEM_ values, averaging −0.97 ± 0.04 MPa, compared to well‐watered (WW), averaging −0.52 ± 0.02 MPa (Table 1). This reduction was also reflected in *g*_s_, where WD plants displayed values of 0.07 ± 0.01 mol m^−2^ sec^−1^, in contrast to WW plants, which maintained an average of 0.21 ± 0.01 mol m^−2^ sec^−1^. π_100_ was measured only once, at the end of the water deficit cycle, without significant differences between water treatments.

**Figure 3:**
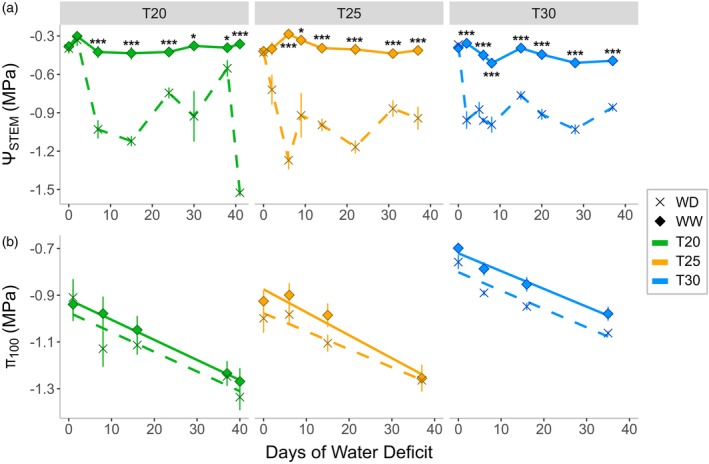
Midday stem water potential (Ψ_STEM_; a) and leaf osmotic potential at full turgor (π_100_; b) measured at different days after the onset of water deficit in greenhouse chambers maintained at daytime temperature of 20°C (T20; green), 25°C (T25; orange) and 30°C (T30; blue) during the 2023 season. Measurements were taken from grapevines subjected to two water regimes: well‐watered (WW; solid line with dots) and water deficit (WD; dashed line with crosses). Markers represent the means; vertical bars indicate ± SE (Ψ_STEM_ *n* = 3–6; π_100_ *n* = 3–5). The lines in A illustrate the seasonal trend, while the lines in B represent the linear regression across all data points within each temperature treatment. Statistical analysis for π_100_ is provided in Table S6. Asterisks indicate significant differences between the treatments on the same date, as determined by ANOVA (*P < 0.05, ***P < 0.001).

In season 2023, water deficit was initiated independently in each greenhouse chamber when plants developed 20 leaves per shoot (Figure S9) and lasted for 35, 37 and 41 days in T30, T25 and T20, respectively. Frequent measurements of stem water potential (Ψ_STEM_) across the different temperature conditions demonstrated consistent trends in deficit intensity: in WW plants, Ψ_STEM_ averaged −0.41 ± 0.03 MPa throughout the period, while in WD plants, Ψ_STEM_ averaged −0.93 ± 0.06 MPa without significant differences between greenhouse chambers (Figure 3). The water deficit induced a reduction in stomatal conductance (*g*_s_) that dropped rapidly to a minimum within the first 8 days of WD, to values below 0.05 mol m^−2^ sec^−1^ corresponding to 5% of the WW controls (Figure S10), which in turn maintained average values along the season of 0.27 ± 0.02, 0.54 ± 0.03 and 0.76 ± 0.02 mol m^−2^ sec^−1^ in T20, T25 and T30, respectively. This initial drop in *g*_s_ by WD was followed by a fluctuating trend maintained below 0.1 mol m^−2^ sec^−1^ during most of the water deficit period (Figure S10). The applied water deficit resulted in an overall shift of π_100_ towards more negative values in WD plants compared to WW plants across all chambers (*P* < 0.001; Table 2; Table S6). Nevertheless, the decrease in π_100_ by WD was relatively low and ranged between 0.07 and 0.09 MPa. On the other hand, the reduction of π_100_ throughout the season was highly consistent and also maintained in WD plants. Indeed, the additional shift of π_100_ towards more negative values observed under water deficit conditions did not affect the rate of seasonal osmotic adjustment, which occurred uniformly across all chambers and was independent of both water deficit and temperature (Figure 3; Table 2; Table S6).

### Osmolyte contribution to the osmotic potential

**Figure 4:**
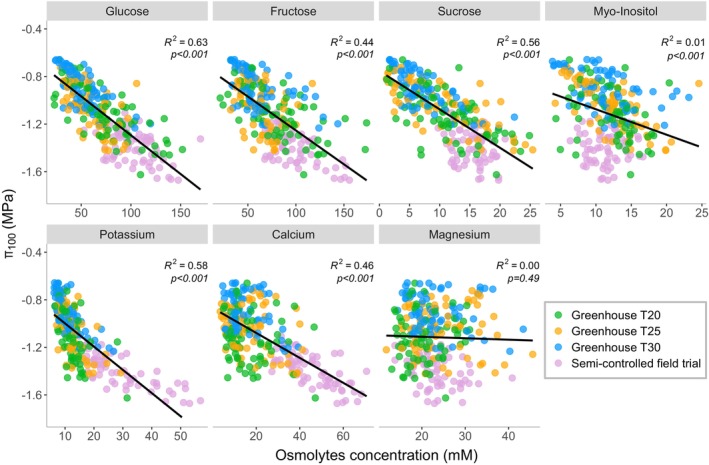
Relationship between the concentration of different osmolytes (mM) and the osmotic potential at full turgor (π_100_) in 2023 across the different experiments. Data points represent original measurements from each temperature: greenhouse chambers set at daytime temperatures of 20°C (T20; green), 25°C (T25; orange), and 30°C (T30; blue), as well as the semi‐controlled field trial exposed to environmental temperature (lilac). The black line indicates the linear regression, which is derived from the combined observations of all sites.

Another objective of this study was to examine the behaviour of the key compounds involved in the osmotic adjustment. To this end, soluble sugars and cations were quantified in the same samples measured with the osmometer. As expected, both cations and soluble sugars contributed to the reduction of π_100_ in all the experiments (Figure 4). Potassium and calcium were significant contributors despite having lower concentrations than sugars. For comparison, the range of concentrations of potassium and calcium was 6.2 to 56.8 mM and 3.5 to 70.7 mM, respectively, while glucose reached up to 170 mM and fructose to 173.6 mM (Figure 4; Figure S11). These sugars were most abundant in the semi‐controlled field trial, which also exhibited the lowest π_100_. As described in Table S8, the total contribution of the analysed osmolytes to π_100_ amounted to an average of 54.5%, ranging from 49.7% to 69.6%. The contribution of soluble sugars to π_100_ across all experimental sites was 40.1%, while the measured cations accounted for an average contribution of 14%. In the semi‐controlled field trial, more negative values of π_100_ than in the greenhouse chambers were observed, because of higher osmolyte concentrations (Figure 4). Here, the contribution of cations to π_100_ gradually increased, rising from 15 to 19.9%, while the contribution of soluble sugars fluctuated between 38 and 46% throughout the season (Table S9). In the greenhouse chambers, we observed a high variability in the contribution of each osmolyte class over time and temperatures (Table S10). Due to collinearity among osmolytes, particularly between glucose and fructose (correlation = 0.92) and potassium and calcium (correlation = 0.86), partial least squares (PLS) analysis was employed to assess the impact of osmolytes on the seasonal osmotic adjustment. The PLS analysis indicated that a single latent component accounted for 84% of the variance in seasonal osmotic adjustment. Based on the *R* ^2^ value and cross‐validation, the model with one component optimized predictive performance, as demonstrated by the lowest RMSEP value (Table S7). In the loadings of the first principal component identified among the osmolytes, glucose was found to be the most significant contributor to explaining seasonal osmotic adjustment, followed closely by potassium, then by fructose and calcium. Glucose and fructose belong to the same class of compounds, indicating a strong influence of sugars on the determination of seasonal osmotic adjustment.

## DISCUSSION

### The rate of seasonal osmotic adjustment is not driven by environmental factors

**Figure 5:**
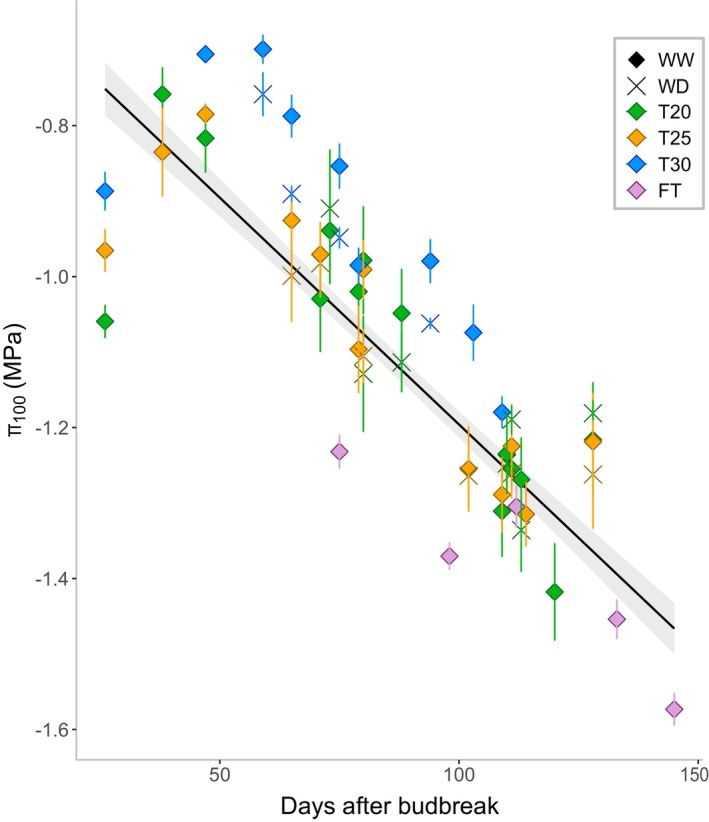
Regression line of osmotic potential at full turgor (π_100_) over Days after budbreak (DAB) across all experimental sites (2022 and 2023). Data were collected under four conditions: day/night temperature of 20/15°C (T20, green), 25/20°C (T25, orange), 30/25°C (T30, blue) and a semi‐controlled field trial (FT; lilac). Well‐watered (WW) plants are represented by rhombus symbols, while water deficit (WD) plants are shown with crosses. Data points indicate mean π_100_ values at each DAB, bars indicate ± SE. The black solid line represents the regression across all markers, and the grey shadow represents the 95% confidence interval. Statistical analysis of the regression is presented in Table S11.

Recent studies have highlighted the ability of grapevines to acclimate to environmental hardening during the vegetative season (Bartlett et al., 2012, 2014; Herrera et al., 2022; Herrera & Hochberg, 2024; Sorek et al., 2021) by adjusting their hydraulic traits, such as leaf osmotic potential (Hochberg et al., 2013, 2017), leaf hydraulic conductance (Martorell et al., 2015), petiole, leaf and stem vulnerability to embolism (Charrier et al., 2018; Sorek et al., 2021) and leaf turgor loss point (Alsina et al., 2007; Herrera et al., 2022; Hochberg et al., 2017). While seasonal osmotic adjustment in grapevine has often been reported in literature (Alsina et al., 2007; Herrera et al., 2022; Herrera & Hochberg, 2024; Sinclair et al., 2024; Sorek et al., 2021), it was not clear whether it was driven by the changing climatic conditions along the season or rather a canopy‐ageing effect. Given that viticulture is most typical in regions where seasonal mean temperatures typically range from 20 to 30 °C, we designed our treatments to reflect these climates documented in different latitudes (Herrera et al., 2018, 2022; Romero et al., 2025; Sinclair et al., 2024). This framework allowed us to compare the seasonal osmotic adjustment of the same genetic material across contrasting environmental contexts, rather than extreme or heatwave conditions. Our results showed a seasonal decrease in the leaf osmotic potential at full turgor (π_100_) that was independent of external factors such as temperature and water availability. Importantly, it was also largely independent of stem water potential. Across 2 years of greenhouse trials, π_100_ consistently became more negative as the season progressed, even under stable conditions of temperature, humidity, photoperiod and soil water content (Figure 2; Figures S4 and S5; Table 2). Similar rates in seasonal osmotic adjustment were observed for both well‐watered (WW) and water deficit (WD) plants, indicating that independently from water availability, grapevines undergo a comparable osmotic adjustment rate along the season (Figure 3). This suggests that seasonal osmotic adjustment is an innate characteristic of the plant rather than only a response to environmental factors. All the π_100_ data collected in different experiments, under diverse environmental conditions and treatments, are consolidated in a single scatter plot (Figure 5). The linear regression analysis of such data reveals that, even if differences in absolute values were present due to environmental conditions, the days after budbreak are the main significant factor affecting the rate of seasonal adjustment (Figure 5; Table S11). This consistency suggests that the seasonal changes in π_100_ are likely pre‐programmed in grapevine plants and are largely environment‐independent. However, variability in our data across specific sampling dates, as well as differences from previous studies, (Alsina et al., 2007; Martorell et al., 2015; Sinclair et al., 2024) indicates that additional factors, such as sampling hour (Gersony et al., 2020), sampled leaf or plant age and the method used (as discussed below), may influence π_100_. Interestingly, our results showed that lower growing temperatures (25 or 20°C) led to more negative π_100_ values compared to 30°C, although they did not influence the rate of osmotic adjustment. The differences in π_100_ reflect the lower sugar concentrations in T30 (Figure S10), likely resulting from stronger carbon sink demand and increased respiration rate (Turner & Jones, 1980). Nevertheless, it is important to recognize that the range of temperatures and stress levels tested in this study did not include extremes. Thus, it remains uncertain whether the same patterns would persist under more extreme conditions, such as temperatures exceeding 30°C, and further studies will be necessary to address this question.

Surprisingly, in our study water deficit induced minimal OA over the entire season (≤0.10 MPa; Figure 3; Table 2; Table S6) and was not significantly influenced by temperature. The limited effect of water deficit on the OA of Pinot noir leaves might be explained in two possible ways. The first is that there is a cultivar‐specific OA behaviour, with different degrees of OA under drought conditions, as suggested for other plant species (Morgan, 1984; Sharma et al., 2023; Turner, 2018). Accordingly, our study shows that Pinot noir exhibited minimal OA (ca. 0.10 MPa), categorising it as a low OA cultivar (or non‐osmoregulatory). Further evidence of cultivar‐specific responses to drought is provided by Schultz (2000), who reported contrasting osmoregulatory strategies in Grenache (osmoregulatory) and Syrah (non‐osmoregulatory). Similarly, Düring (1984) observed a strong OA in Silvaner but minimal adjustment in Riesling. These findings emphasize the importance of considering cultivar‐specific effects when assessing OA to drought in grapevines.

Another possible explanation for the differences in OA between studies might be related to the intensity of the water deficit experienced by the plants. In our experiments, the water deficit applied was moderate (Ψ_STEM_ from −0.8 to −1.2 MPa), enough to downregulate *g*_s_, but not enough to maintain the stomata fully closed for the entire water deficit periods. Patakas et al. (2002) observed an osmotic potential difference of 0.41 MPa between control and drought‐stressed plants over 10 days, but the drought‐stressed plants in that study experienced higher water stress intensities than in ours, with predawn Ψ values reaching −1.2 MPa as compared with −0.3 MPa in the control. Similarly, Hochberg et al. (2013) reported an OA of ca. 0.32 MPa in response to water potentials lower than those observed in our study, while Rodrigues et al. (1993) reported a value of approximately 0.45 MPa. Further, many studies have documented substantially lower Ψ_STEM_ values (<−1.5 MPa) than those reported in this experiment (> − 1.2 MPa) without observing any wilting (de Souza et al., 2005; Martínez et al., 2016; Schultz, 2003), suggesting a larger degree of OA in those cases. This would suggest some degree of OA in response to water deficit, presumably with the objective of maintaining growth (Schultz & Matthews, 1993). However, in our experiments, the magnitude of this adjustment appears small relative to the innate seasonal adjustment, highlighting that the effects of water deficit are complex and factors such as genotype (Abrams, 1988b; Roberts et al., 1980), the growth stage at the time of stress (Roberts et al., 1980) and the speed and intensity of stress exposure (Abrams, 1988b; Blum, 2017; Turner & Jones, 1980) ultimately shape the plant's adaptive response.

### Contributors to osmotic adjustment

Over time, grapevine leaves accumulated sugars and cations, leading to a reduction in π_100_. Partial least squares (PLS) analysis in our study identified soluble carbohydrates (mainly glucose and fructose) and inorganic ions (primarily potassium and calcium) as the key contributors to OA in grapevine leaves. The predominance of soluble carbohydrates as the main component of π_100_ aligns with previous findings in oak trees (Epron & Dreyer, 1996; Gersony et al., 2020) and grapevine (Patakas, 2000; Rodrigues et al., 1993). However, we observed a lower contribution (33–57%) compared to the ca. 59–72% reported by Patakas (2000) and a greater influence of glucose and fructose rather than the high sucrose values reported by Rodrigues et al. (1993). Methodological considerations are also crucial for interpreting our results. In our study, we chose not to assume a constant apoplastic‐to‐symplastic ratio in our samples to calculate π_100_, an approach that yielded more diluted π_100_ values compared to pressure–volume curve‐derived ones and, therefore, slightly less negative than those reported in the literature for grapevines. However, by using the same sap sample in the osmometer and for the quantification of osmolyte concentrations, we can better relate to the osmolytes contributing to π_100_, minimizing the variability introduced by metabolite extraction processes. Cation concentration also increased significantly over the season, consistent with prior studies (Degu et al., 2019; Patakas et al., 2002), which identified potassium and calcium as key contributors to OA. However, their contribution to π_100_ (12–20%) was lower than that of soluble sugars. Overall, the combined impact of these compounds on π_100_ ranged from 52 to 69.6%, confirming their crucial role in OA. Notably, neither sugars nor cations exhibited a strictly consistent contribution or uniform trend over time or across experiments. Therefore, instead of identifying a single dominant factor driving seasonal osmotic adjustment or the response to water deficit, our results suggest a general pattern of solute accumulation throughout the season. This aligns with previous findings showing seasonal fluctuations in the contribution of soluble sugars (Patakas et al., 2002) and their concentration (Gersony et al., 2020), as well as responses to water deficit (Perry et al., 2025).

Taken together, our results confirm that there is a seasonal osmotic adjustment in grapevine where solute concentrations increase over time, resulting in lower π_100_ values. Such OA is not the result of temperature increases or water deficit periods occurring throughout the season, although heat and drought spells may impact the absolute π_100_. Furthermore, OA seems not to be driven by the accumulation of a single dominant compound but rather by a complex accumulation of solutes that includes soluble sugars (mainly glucose and fructose) and cations (calcium and potassium). These findings suggest that grapevines may have evolved a pre‐programmed seasonal osmotic adjustment strategy, which operates largely independently of environmental variability. Environmental constraints such as drought can induce a faster OA under certain conditions (e.g. surpassing critical thresholds of Ψ) as evident from other studies and species (Bowman & Roberts, 1985; Sorek et al., 2023). In our multi‐year study, we did not observe a change in π_100_ in response to mild water deficit comparable to the changes driven by the seasonal progression. Therefore, it is important to study OA in a seasonal context to accurately interpret the grapevine's active engagement in this process and avoid misinterpretations of the response (Jacobsen et al., 2007; Turner & Jones, 1980). It is important to note that the findings of this study are probably not unique to grapevines. Multiple plant species from temperate climates exhibit seasonal plasticity in drought tolerance (Abrams, 1988b; Bartlett et al., 2012; Bowman & Roberts, 1985; Feng et al., 2023; Jacobsen et al., 2007; Kolb & Sperry, 1999; Sorek et al., 2022), and seasonal osmotic adjustment might be involved as part of a complex coordinated series of physiological changes. Finally, while the results of our experiments are conclusive within the range of environmental conditions studied, they also highlight the need to further investigate plant behaviour under more extreme situations (e.g. broader temperature conditions, severe drought, changes in VPD) and to elucidate other potential regulatory factors, such as light and photoperiod, to fully understand the role of OA in plant–environment interactions.

## MATERIALS AND METHODS

### Experimental design and plant growth conditions

Three trials were conducted on a total of 72 potted *Vitis vinifera* cv. Pinot noir, grafted onto SO4 rootstock. Between January and May of 2022 and 2023, greenhouse experiments were set out to study the impact of temperature (T) and water deficit (WD) on the seasonal changes of leaf osmotic potential π_100_ (Figure 1a,b). Greenhouse experiments were complemented during the summer of 2023 by sampling leaves from a semi‐controlled field trial to track π_100_ along the season (Figure 1c). In all experiments, pots were filled with a commercial substrate (CL ED73 Einheitserde, Sinntal, Germany) supplemented with 25% perlite.

### Greenhouse experiment

In the first semesters of 2022 and 2023, 48, 4‐year‐old grapevines planted in 12 L pots were grown in greenhouses with controlled environmental conditions. All the plants were grown under homogeneous conditions until they had seven leaves (acclimation period); the greenhouse chamber was set to 20/15°C day/night, constant air relative humidity of ca. 40% and 12 h (7:00–19:00) supplemental artificial light (400 μmol m^−2^ sec^−1^; Philips Master PI‐T Plus 400W/645 E40). After the acclimation period, temperature differentiation was imposed (described below) by separating the plants into two and three different chambers in 2022 and 2023, respectively. In each chamber, plants were arranged in four rows. Two (in 2022) and four shoots (in 2023) per plant were trained vertically with the aid of bamboo sticks and hanging threads (Figure S1); every shoot was trimmed at 16 nodes in 2022 and 20 nodes in 2023. Lateral shoots were allowed to grow, while all the inflorescences were removed as soon as they differentiated. All the plants were irrigated daily to soil capacity using three pressure‐compensated drippers (Netafim PCJ 2 L h^−1^) per pot.

### Temperature differentiation

In 2022, when plants reached 7 leaves per stem, temperature differentiation between the two chambers was imposed over 10 days, starting on January 14th, corresponding to the day after budbreak (DAB) 38. The temperature was increased in one of the chambers by +1°C every 2 days, with adjustments made on January 16th, 18th, 20th, and 24th. Thereafter, chamber conditions remained constant with the only difference between them in temperature regimes set at 20/15°C (T20) and 25/20°C (T25) day/night. At the end of the 2022 experiments (ca. June 2022), all plants were stored in a dark cooling room at 5°C to overwinter.

In 2023, the same plants used in 2022 were utilized. An additional temperature regime was incorporated into the experimental design, and therefore three greenhouse chambers were set with day/night temperature settings of 20/15°C (T20), 25/20°C (T25) and 30/25°C (T30). Three groups of 16 plants each were formed from the original set, as illustrated in Figure S2; eight plants from the T20 and T25 chambers used in the previous year were transferred to the newly created chamber while maintaining the same water regime as in the previous year. Before temperature differentiation, every shoot was marked to recognize leaves formed during acclimation and after temperature differentiation.

### Water‐deficit imposition

In each of the greenhouse chambers, half of the plants were subjected to water‐deficit (WD) treatments by removing two out of the three drippers per pot. Therefore, WD plants received one‐third of the irrigation volume of the well‐watered (WW) ones. As the varying temperatures in different greenhouse chambers led to different water demands (Figures S3 and S4; Table S1) and different growth rates (Figure S7), the water treatment was standardized based on the control plants. Water regimes in each greenhouse chamber and in the semi‐controlled field trial were systematically adjusted to maintain the midday stem water potential (Ψ_STEM_) of WW plants above −0.50 MPa throughout the entire experimental season (Table S2). In 2022, the WD treatment was imposed in both chambers simultaneously when all plants had at least 16 nodes per shoot. WD treatment was maintained for 51 days in both chambers. In 2023, WD started separately in each chamber and was imposed when all plants in the given chamber reached 20 nodes per shoot, as indicated in Figure S9. The WD duration was designed to last from 35 to 40 days. As WD started at different times in the different chambers, to facilitate comparisons, we utilize the term days of water deficit (DOW). After the WD treatment had finished, all plants were manually rewatered to saturation, and the three drippers were repositioned in each pot.

### Semi‐controlled field experiment

To complement the observations in the greenhouse regarding seasonal osmotic adjustment, we also monitored leaves from potted grapevines growing outside in field conditions under a rain shelter as described in (Herrera et al., 2024). A total of 24 Pinot noir grapevines, 5 years old and potted in 43 L plastic pots, were available and growing in the experimental field of the BOKU University campus in Tulln, Austria (48.32° N, 16.07° E). The plants were arranged within three rows oriented North–South and trained with a vertical shoot position with the aid of a trellis system (Figure S1). Plants were cane pruned, keeping six buds in the fruiting cane and one renewal spur with two buds. In summer, shoots were trimmed once when they extended beyond the trellis (i.e. canopy higher than 1.4 m). Plants were maintained under well‐irrigated conditions during the whole season by daily irrigating them to field capacity during night‐time using an automatic drip irrigation system (four drippers/pot).

### Treatments monitoring

During the experiments, temperature and relative humidity were recorded in the greenhouse chambers and semi‐controlled field experiments on an hourly basis. Exhaustive information regarding temperature, heat accumulation and trends is provided in the Supplementary Material (Figures S3–S6). Plant phenology was monitored from budbreak onward by recording the number of nodes per shoot per plant weekly in greenhouse experiments. In the semi‐controlled field trial, phenology was assessed based on the main developmental stages. To standardise observations across experiments, time was expressed as days after budbreak (DAB) (Figure S9).

Plant water status was monitored weekly by measuring the midday stem water potential (Ψ_STEM_) on fully expanded leaves on 3–6 plants per treatment using a Scholander chamber between 12:00 and 14:00, as described by Levin et al. (2019). Leaf stomatal conductance (*g*_s_) was measured in greenhouse experiments with a LI‐600 Porometer/Fluorometer (LI‐COR Biosciences Inc., Lincoln, NE, USA). Fully expanded leaves from the main shoot with PAR >90 μmol m^−2^ sec^−1^ every 2–6 days were measured between 09:30 and 12:30.

### Leaf sampling for osmotic potential at full turgor (π_100_)

In all experiments, leaves were sampled throughout the trials to track changes in the osmotic potential at full turgor. The sampling strategy is graphically shown in Figure 1. Only one leaf per plant was sampled at each time point for these analyses, ensuring independence of observations at the plant level. To minimise diurnal variability (Bowman & Roberts, 1985), the sampling for π_100_ was consistently taken at the same time of day across all sampling dates, ensuring comparability of seasonal trends within our study. To ensure that physiological measurements captured the developmental progression in both contexts, sampling in the greenhouse and semi‐controlled field experiment was conducted on leaves that developed at slightly different times due to variations in leaf availability and growth rates.

To track seasonal osmoregulation, we collected 4–9 basal leaves (3rd to 8th nodes) of WW plants from each greenhouse chamber ca. once a month (temporal scale expressed as days after budbreak; DAB). In the semi‐controlled field trial, 11–22 leaves were sampled on 5 sampling dates between July and September from the West side between the 7th and the 10th internode.

To evaluate the effect of water deficit on the osmotic potential, we sampled leaves from the apical zone of the canopy (between 10th and 15th internode, i.e. leaves formed after temperature differentiation). In 2022, only the end of the WD treatments was considered (DOW 40), while in 2023, samplings were done at DOW 0, 6, 14–15 and 35–37–40. At each sampling date, one fully expanded and healthy leaf per plant was collected between 8:00 and 9:30 am from 4 to 6 different plants per treatment in the greenhouse trials and from 11 to 22 in the semi‐controlled field trial. Leaves were wrapped in plastic film and then excised with the petiole, which was immediately placed inside a 2 mL microtube containing distilled water to allow the leaf to rehydrate for 4–5 h in a dark box in the laboratory. After rehydration, the petiole was cut off, and the leaf lamina, still wrapped in plastic foil, was wrapped in aluminium foil and immediately snap‐frozen in liquid nitrogen and stored at −80°C. Leaves were let to thaw at ambient temperature inside the plastic foil to avoid water condensation on the lamina. Once completely thawed (after ca. 5 min), the leaf lamina was collected, excluding the main veins, and placed into a centrifugal filter (Corning Costar® Spin‐X® centrifuge tube filters with 0.45 μm cellulose‐acetate membranes, Corning Incorporated) inserted in a 2‐mL Eppendorf tube. The tube lid was then sealed with parafilm to prevent evaporation. The samples were then centrifuged at 17 000 ***g*** for 20 min at 18°C to extract the leaf sap, and an aliquot of 10 μL was pipetted onto a filter paper disc to measure the osmolality in a vapour pressure osmometer (VAPRO 5600, ELITechGroup, Utah, USA). Osmolarity (mmol L^−1^) was obtained assuming water density at 1000 kg m^−3^ (Rasouli, 2016). Osmotic potential (MPa) was calculated according to the Van't Hoff equation (Equation 1).

(1) π=−CRT

where *C* is the solute concentration (in mol L^−1^), *R* is the universal gas constant (0.00831 MPa L mol^−1^ K^−1^), and *T* is the working temperature of the osmometer (K).

The π_100_ measurements included both apoplastic and symplastic water since the values were not adjusted with a theoretical apoplastic water fraction. The rest of the sap was stored at −80°C until further analysis of osmolytes.

### Osmolytes quantification and contribution to osmotic potential

Leaf sap was analysed by ion chromatography to quantify the concentration of soluble carbohydrates and cations, to assess their contribution to the sap osmotic potential previously measured with the osmometer.

The sap was thawed at ambient temperature, properly diluted with MilliQ water and then filtered using a 0.45‐μm pore size nylon syringe filter. A high‐performance liquid ion‐chromatography system (Dionex ICS‐5000+ DC, Thermo Scientific) was used for the analysis of soluble carbohydrates (by pulsed amperometric detection) and cations (by conductivity detection). Carbohydrates were analysed as described by Savi et al. (2019) using a CarboPac PA20 column 3 × 150 mm, with NaOH 52 mM as eluent with isocratic separation conditions at 30°C and a flow rate of 0.5 mL min^−1^ for 40 min per sample. Cations were separated using an IonPac CS12A column 4 × 250 mm, using methanesulfonic acid (20 mN) as eluent with isocratic separation conditions at 25°C with a flow rate of 1 mL min^−1^ for 15 min per sample. Metabolites were identified and quantified by comparison with analytic standards of glucose, fructose, sucrose, myo‐inositol (Sigma‐Aldrich) or Mg, K, and Ca (Thermo Scientific) using Chromeleon 7 software. The carbohydrate analysis method allows the separation and quantification of more compounds than the ones presented and discussed here (Figures S7 and S8); however, sample dilutions based on the overwhelming abundance of glucose, fructose, and sucrose in all samples, resulted in a low resolution of the other carbohydrates. Similarly, cations below the limit of quantification (Na, Li) were not considered in the analysis.

The concentration of the different cations and carbohydrates was used to calculate their osmotic potential by using the Van't Hoff (Equation 1); thereafter, we calculated the contribution of each metabolite to the leaf sap osmotic potential by dividing the results by the osmometer values.

### Statistical analysis

Statistical analyses and graphical representations were conducted using R (R Core Team, 2021). To examine the effects of time, temperature and water regime on π_100_, linear models were fitted using the lm function from the stats package (R Core Team, 2021) as reported in Tables S4–S7 and S11. The significance of the model coefficients was evaluated using *P*‐values based on *t*‐tests for each coefficient. Additionally, the analysis of variance (ANOVA) was conducted to assess the contribution of each predictor to the variation in π_100_, with the anova function from the same package. For analyses focused on π_100_ on specific dates (e.g. Greenhouse 2022), one‐way or two‐way ANOVA tests were employed, depending on the experimental design. After performing an ANOVA test, we conducted a post hoc Tukey's HSD test using the emmeans package (Lenth, 2025) to compare differences between temperature groups while controlling for multiple comparisons.

Linear models were also fitted to investigate the impact of individual osmolytes on π_100_. To further explore the relationship between π_100_ and the concentration of the different osmolytes, a partial least squares (PLS) regression analysis was performed using the pls package (Liland et al., 2024). To prevent disproportion between the osmolytes, they were scaled using Z‐score scaling, transforming each variable to have a mean of 0 and a standard deviation of 1. Cross‐validation was used to assess model performance, along with metrics such as root mean squared error of prediction, mean squared error of prediction, and *R* ^2^.

## AUTHOR CONTRIBUTIONS

JCH acquired the research funds. EF and JCH designed the experiments. EF executed the experiments and the samplings with the support of AK and SLT. EF, AK and FDB measured the osmotic potentials and analysed the osmolytes under the supervision of JCH. EF analysed and interpreted the data and drafted the manuscript under the supervision of JCH. UH and GAG contributed to the discussion of the results and edited the manuscript. AF critically reviewed the manuscript.

## CONFLICT OF INTEREST

The authors declare no conflict of interest.

## Supporting information


**Figure S1.** Pictures of the semi‐controlled field trial and greenhouse trials.
**Figure S2.** Experimental set‐up in the greenhouse.
**Figure S3, S4, S6.** Greenhouse 2022, 2023 and semi‐controlled field climate data.
**Figure S5.** Growing degree days semi‐controlled field trial and greenhouse 2022, 2023.
**Figure S7, S8.** Example chromatograms of carbohydrates and cations.
**Figure S9.** Greenhouse 2023 shoot growth.
**Figure S10.** Stomatal conductance greenhouse 2023.
**Figure S11.** Total concentration of sugars and cations in greenhouse 2023.
**Table S1.** Irrigation plan greenhouse experiments in 2022 and 2023.
**Table S2.** Average midday stem water potential semi‐controlled field.
**Tables S3–S11.** Statistical results.
**Tables S8–S10.** Average calculated contribution of individual osmolytes.

## Data Availability

The data that support the findings of this study are openly available in ‘Leaf seasonal osmotic adjustment in grapevine’ in the Zenodo repository at https://zenodo.org/records/16993248.
