# Supplementary material for: Leaf seasonal osmotic adjustment is not driven by temperature or water deficit
Source: Plant J. 2025 Nov 14;124(3):e70569. doi: 10.1111/tpj.70569 (PMC12617389; doi:10.1111/tpj.70569)
Supplement: Supplementary file 1 — Figure S1. Pictures of the semi‐controlled field trial and greenhouse trials. Figure S2. Experimental set‐up in the greenhouse. Figure S3, S4, S6. Greenhouse 2022, 2023 and semi‐controlled field climate data. Figure S5. Growing degree days semi‐controlled field trial and greenhouse 2022, 2023. Figure S7, S8. Example chromatograms of carbohydrates and cations. Figure S9. Greenhouse 2023 shoot growth. Figure S10. Stomatal conductance greenhouse 2023. Figure S11. Total concentration of sugars and cations in greenhouse 2023. Table S1. Irrigation plan greenhouse experiments in 2022 and 2023. Table S2. Average midday stem water potential semi‐controlled field. Tables S3–S11. Statistical results. Tables S8–S10. Average calculated contribution of individual osmolytes. [file TPJ-124-0-s001.docx]

**Leaf seasonal osmotic adjustment is not driven by temperature or water deficit**

Elena Farolfi*, Adéla Kulhánková, Federica De Berardinis, Soma László Tarnay, Gregory A. Gambetta, Uri Hochberg, Astrid Forneck, Jose Carlos Herrera

* Correspondence: elena.farolfi@boku.ac.at

**SUPPLEMENTARY MATERIAL**

**1. Pictures of experimental setups**

**
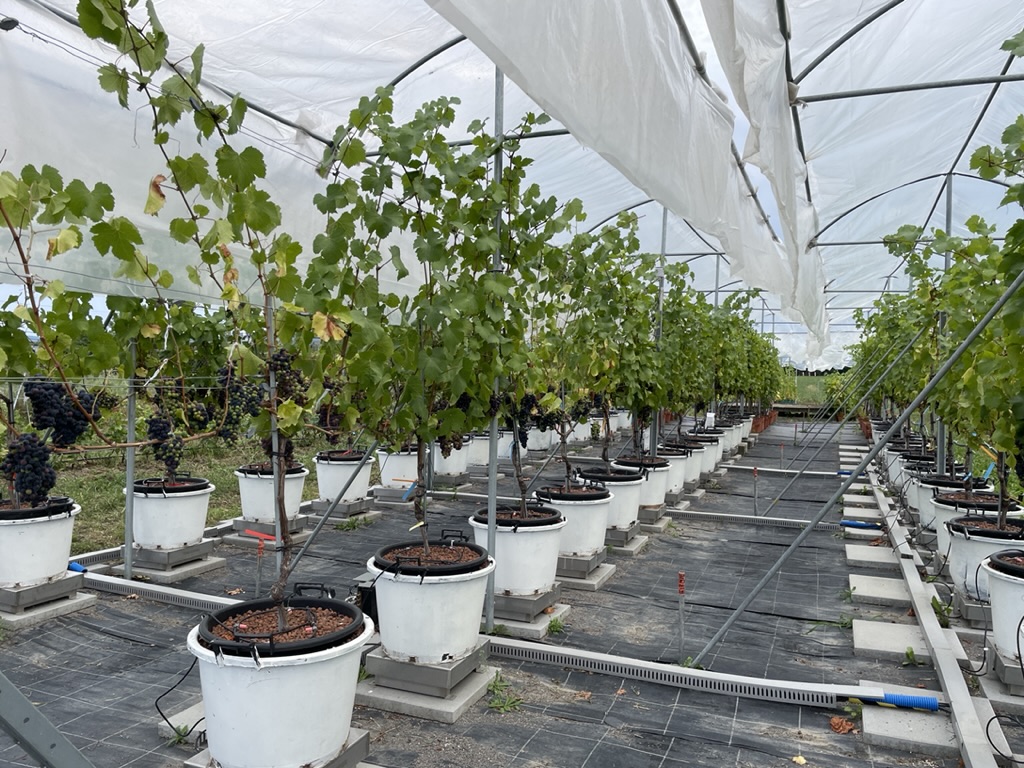

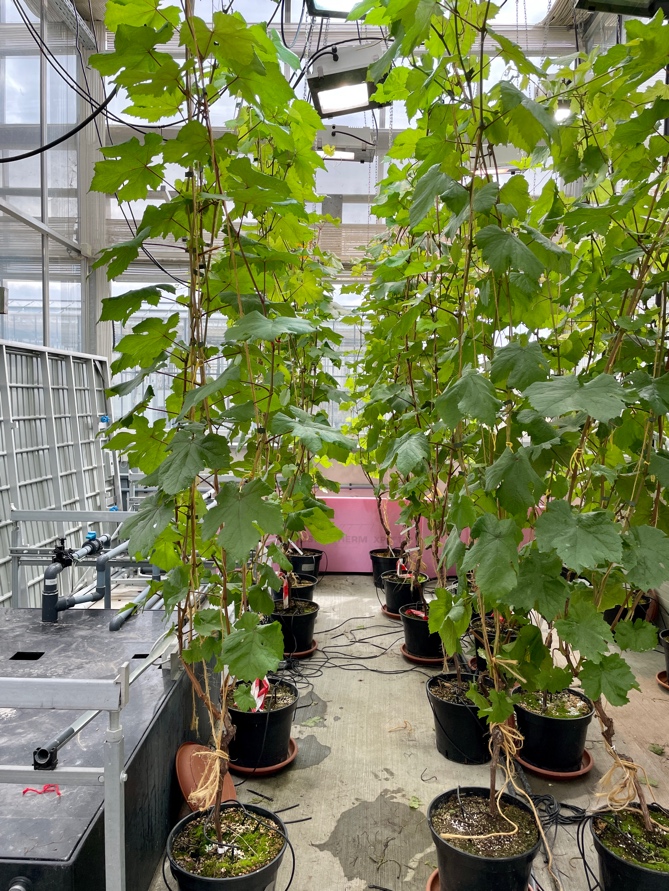
**

Greenhouse trial

Semi-controlled field trial

**Fig. S1** – Pictures of the semi-controlled field trial and greenhouse trials. In the semi-controlled field trials, plants were arranged in three North-South oriented rows and trained using a vertical shoot positioning system with the aid of a trellis. In the greenhouse trials, plants were arranged in four rows across three different chambers with three different temperature regimes, and they were grown vertically with the aid of hanging threads.

**2. Design of experimental setups**


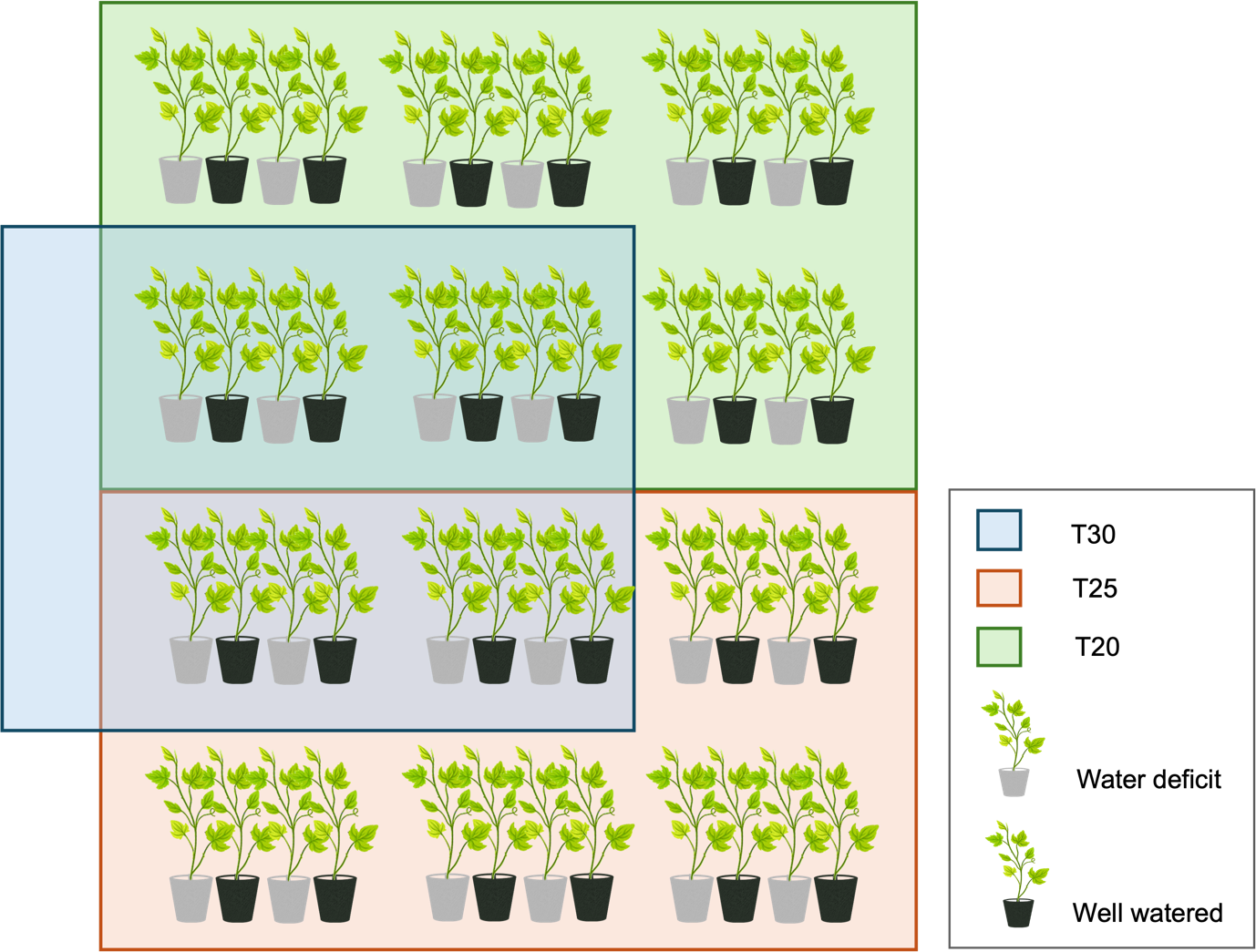


**Figure S2** – Experimental set-up in the greenhouse. The same plants were used in 2022 and 2023. In 2022, only two chambers were used: T20 (green; set at 20/15°C day/night) and T25 (orange; 25/20°C day/night). In 2023, an additional temperature regime was incorporated into the experimental design: T30 (blue; 30/25°C day/night). The plants for T30 were randomly selected from T20 and T25 (8 plants per chamber, 50% well-watered and 50% water deficit).

**3. Field and greenhouse environmental conditions**

To visualise the temperature variations during the experimental period in the greenhouse, heat accumulation was calculated using the Growing Degree Day (GDD) model, starting from the day of budbreak for each experiment. Daily GDD values were summed over the study period to calculate cumulative GDD, providing a measure of heat units reached within the period. GDD has been calculated as in eq (a):

Equation a:

$$\text{GDD= }\sum\left( \frac{\text{Tmax+Tmin}}{\text{2}} \right)-10$$

Where Tmax​ represents the daily maximum temperature (°C), Tmin​ is the daily minimum temperature (°C), and 10°C was set as the base temperature, in line with the phenological threshold for grapevines (Winkler et al., 1974).

The temperature in the semi-controlled field trial had an average of 21°C, an average of Tmax 27°C, and an average Tmin of 8°C. As reported in Fig. S2 there were temperature fluctuations during the period considered: from day after budbreak (DAB) 103 to 112 Tmax was lower than 23°C, while in the period DAB 115 to 127, Tmax was over 30°C. The accumulation of Growing Degree Days (GDD), as shown in Figure S3A, increased from 420°C on the first sampling date to 1167°C on the last sampling date.

In the greenhouse chambers, although set to a constant temperature, some fluctuations were observed in all the chambers in both years. In 2022, the average diurnal temperature was 21°C for T20 and 25°C for T25 (Fig S3), resulting in GDD values at the last sampling of 1325°C and 1693°C, respectively, over 128 DAB and an average daily VPD of 1.6 and 2.0 kPa. In 2023, temperatures in the different chambers had fluctuations along the experimental season, but still they remained distinctly differentiated, as shown in Figure S4, with average diurnal temperatures of 20.8°C, 24.6°C, and 28.3°C for T20, T25, and T30, respectively. Average diurnal VPD was 1.4, 1.6 and 2.2 kPa for T20, T25, and T30, respectively. Despite the fluctuations, differences in temperatures are reflected in the GDD accumulated at the end of the season with values corresponding to 856, 1246°C, and 1504 °C in T20, T25, and T30, respectively.


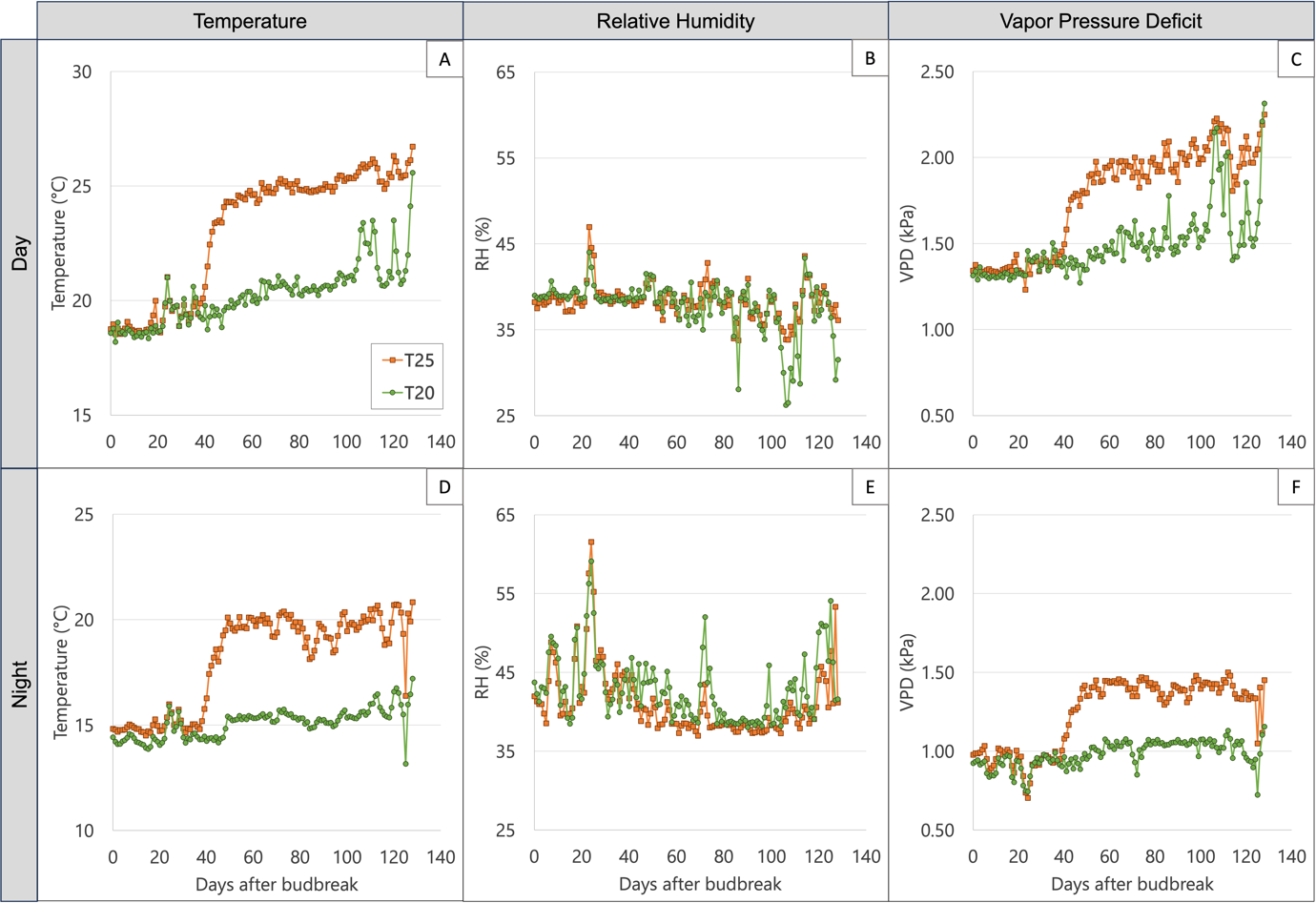


**Figure S3** – Daily average temperature (T, a and d), relative humidity (RH, b and e), and vapor pressure deficit (VPD, c and f) during the day (7:00 to 18:59; a, b, c) and night (19:00 to 06:59; d, e, f) in the year 2022 in the greenhouse trial, expressed in days after budbreak. Different colors represent different greenhouse chambers (Green = T20, Orange = T25). Markers represent the daily means.


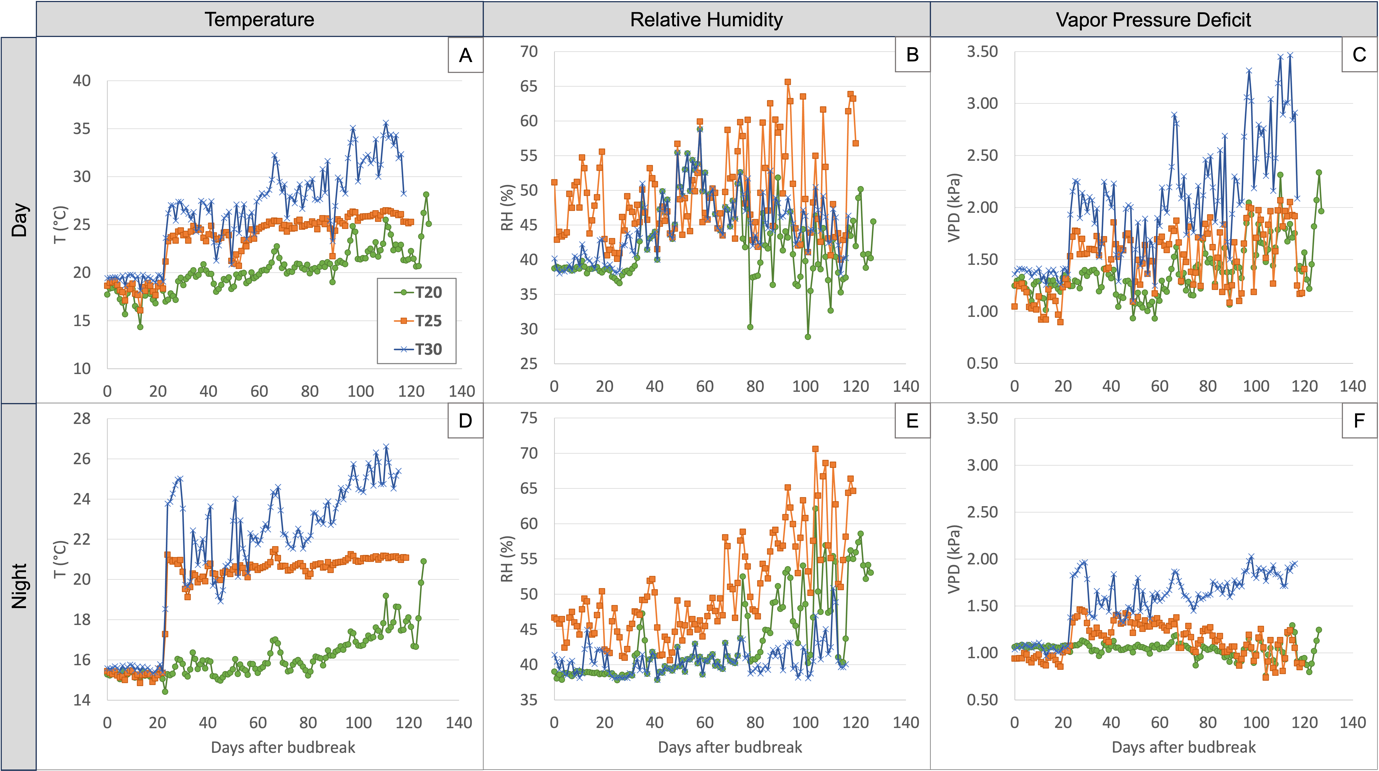
**Figure S4** – Daily average temperature (T, a and d), relative humidity (RH, b and e), and vapor pressure deficit (VPD, c and f) during the day (7:00 to 18:59; a, b, c) and night (19:00 to 06:59; d, e, f) in the year 2023 in the greenhouse trial, expressed in days after budbreak. Different colors represent different greenhouse chambers (Green = T20, Orange = T25, Blue = T30). Markers represent the daily means.

**Table S1** – Irrigation plan for well-watered (WW) plants (in L/day per pot) at different days after bud break (DAB) in the greenhouse experiments in 2022 and 2023.

|  |  | **Days after budbreak (DAB)** | | | | | | | | | | | |
| --- | --- | --- | --- | --- | --- | --- | --- | --- | --- | --- | --- | --- | --- |
| **Greenhouse 2022** |  | 45 | 49 | 64 | 66 | 69 | 72 | 73 | 76 | 83 | 87 | 94 |  |
| T20 |  | 0.3 | 0.3 | 0.3 | 0.4 | 0.8 | 0.6 | 0.5 | 0.4 | 0.5 | 0.6 | 0.7 |  |
| T25 |  | 0.5 | 0.4 | 0.5 | 0.7 | 1.2 | 1.0 | 0.9 | 0.7 | 0.8 | 0.9 | 1.0 |  |
|  |  |  |  |  |  |  |  |  |  |  |  |  |  |
|  |  | **Days after budbreak (DAB)** | | | | | | | | | | | |
| **Greenhouse 2023** |  | 37 | 39 | 47 | 54 | 66 | 69 | 73 | 74 | 79 | 81 | 94 | 96 |
| T20 |  | 0.7 | 0.7 | 1.0 | 1.0 | 1.2 | 1.2 | 0.8 | 0.6 | 0.9 | 1.1 | 1.2 | 1.2 |
| T25 |  | 1.0 | 1.0 | 1.4 | 1.4 | 1.4 | 1.4 | 1.2 | 1.2 | 1.4 | 1.7 | 1.5 | 1.5 |
| T30 |  | 1.3 | 1.1 | 1.6 | 1.5 | 1.8 | 1.7 | 1.4 | 1.4 | 1.7 | 1.9 | 1.7 | 1.7 |
|  |  |  |  |  |  |  |  |  |  |  |  |  |  |


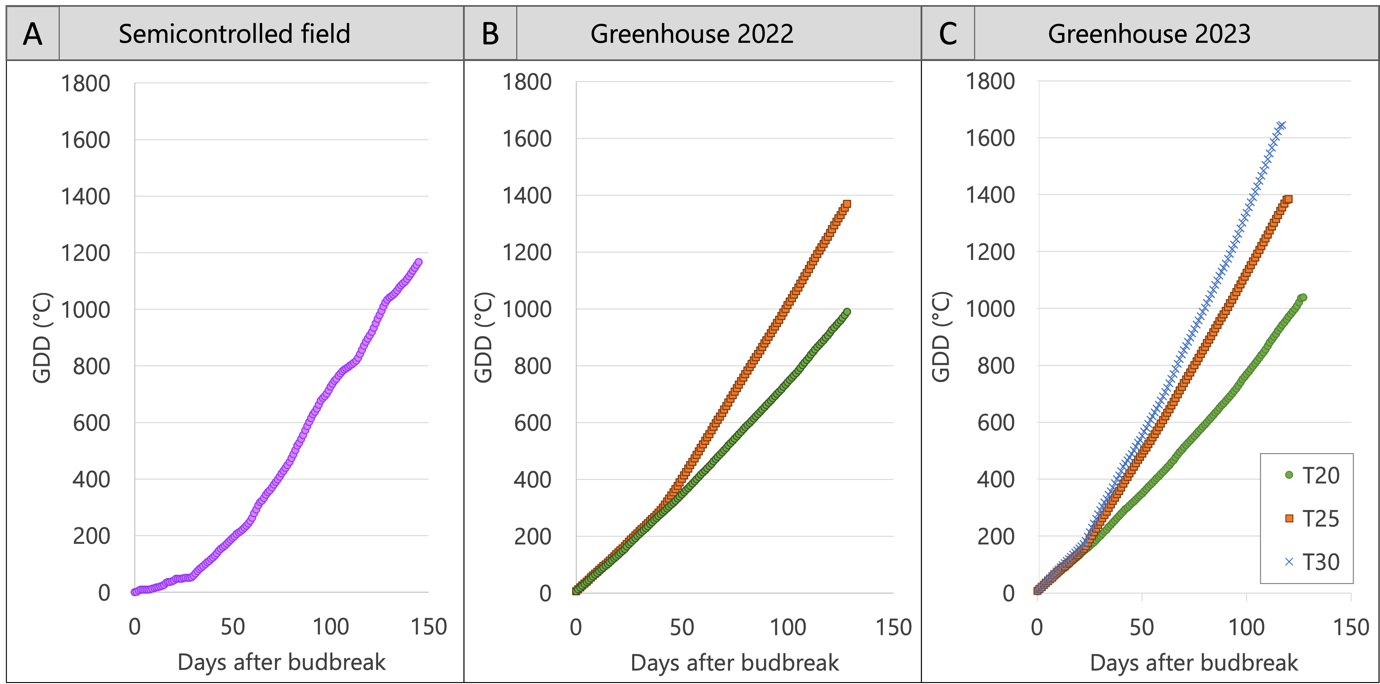
**Figure S5** – Growing Degree Days accumulated from budbreak in semi-controlled field (A), greenhouse 2022 (B) and greenhouse 2023 (C). Markers represent the accumulated GDD on a specific day after budbreak. Different colors represent different greenhouse chambers (Green = T20, Orange = T25, Blue = T30).


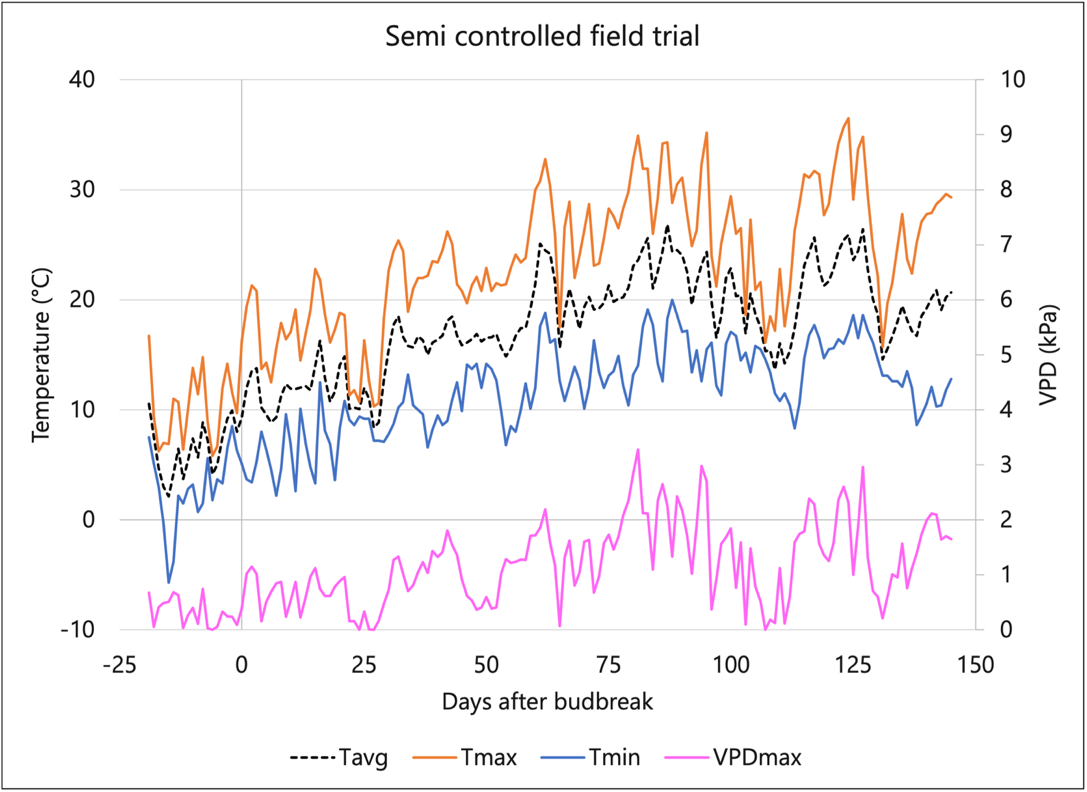


**Figure S6** - Semi controlled field trial daily maximum (Tmax, orange line), minimum (Tmin, blue line), and average (Tavg, black dotted line) temperatures and daily maximum VPD (kPa; VPDmax, magenta line) calculated as the average during the central hours of the day from 11 am to 3 pm during the experiments.

**Table S2** – Average midday stem water potential (Ψ_STEM_; MPa) at different days after budbreak (DAB) of Pinot noir grapevines growing in the semi-controlled field trial. Values are averages with relative standard deviation (St.Dev).

| DAB | Ψ_STEM_ | n |
| --- | --- | --- |
| 98 | -0.50 ± 0.07 | 24 |
| 102 | -0.48 ± 0.05 | 12 |
| 112 | -0.35 ± 0.04 | 12 |
| 116 | -0.44 ± 0.05 | 12 |
| 119 | -0.33 ± 0.04 | 12 |
| 124 | -0.36 ± 0.03 | 12 |
| 127 | -0.35 ± 0.02 | 12 |
| 133 | -0.39 ± 0.03 | 12 |
| 139 | -0.43 ± 0.04 | 12 |
| 145 | -0.41 ± 0.07 | 12 |

**
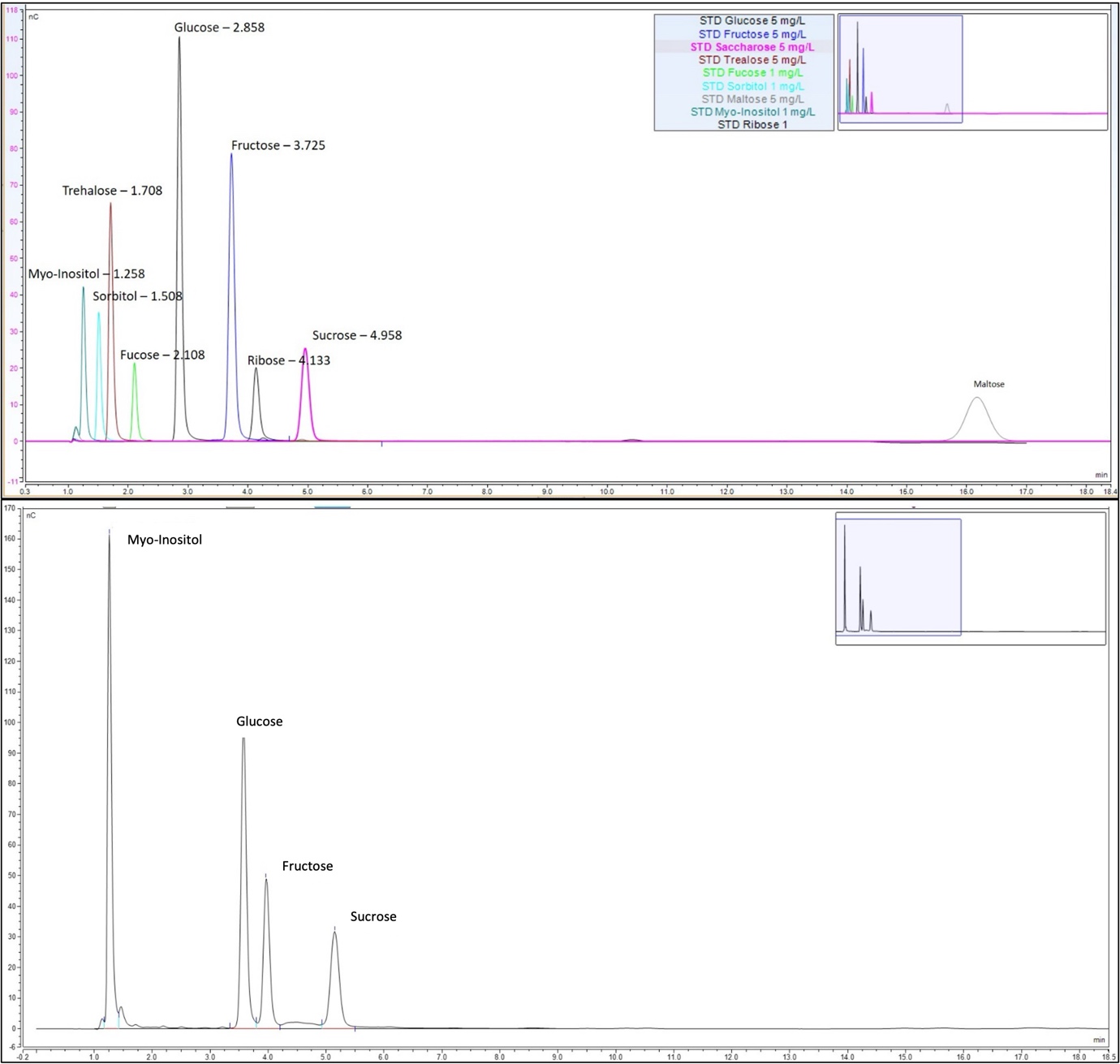
**

**Figure S7** – Chromatograms showing the standard used for carbohydrate identification alongside an example chromatogram from a leaf sap sample.

**
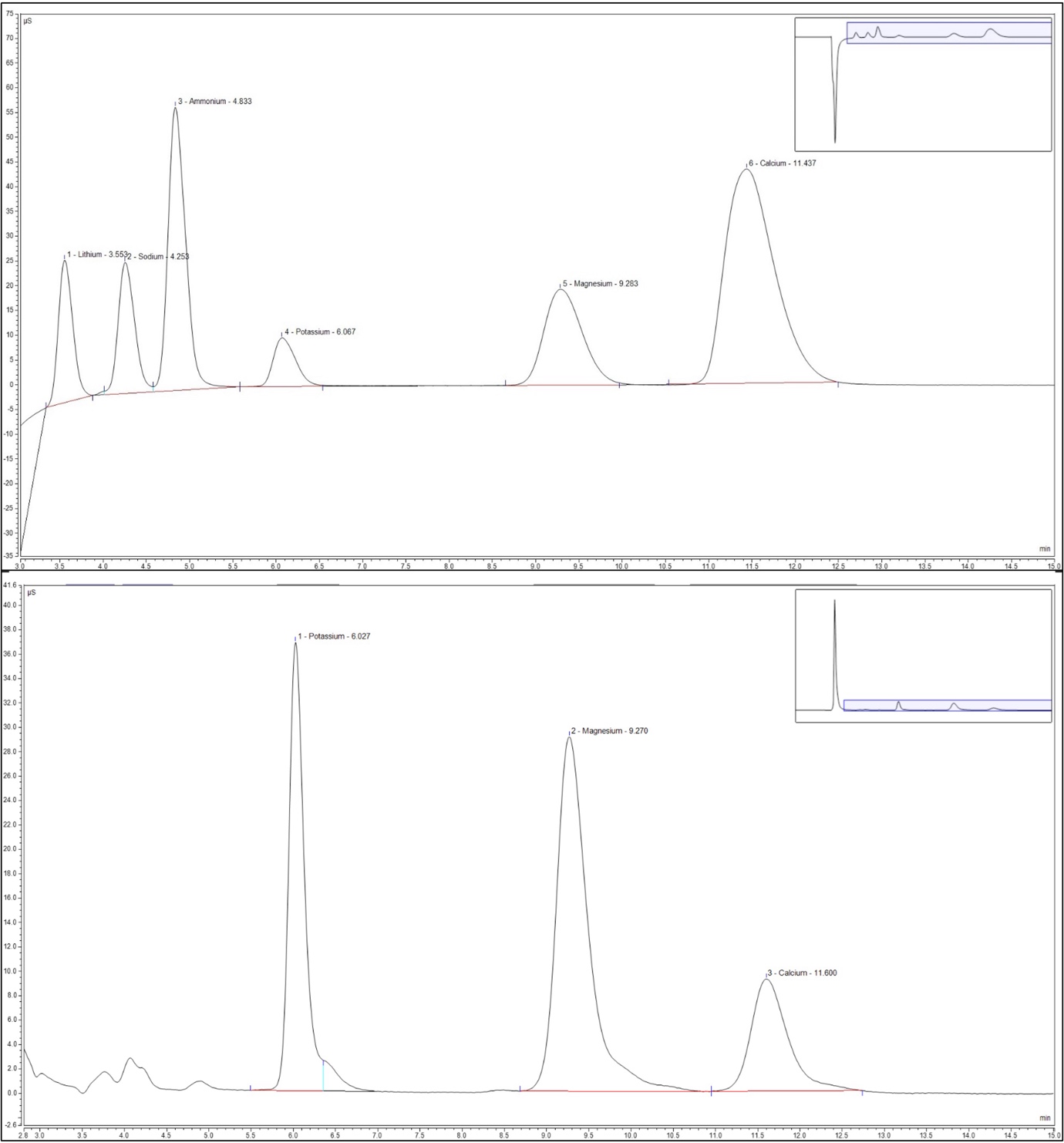
**

**Figure S8** – Chromatograms showing the standard used for cation identification alongside an example chromatogram from a leaf sap sample.

**4. Plant phenology in different greenhouse chambers**


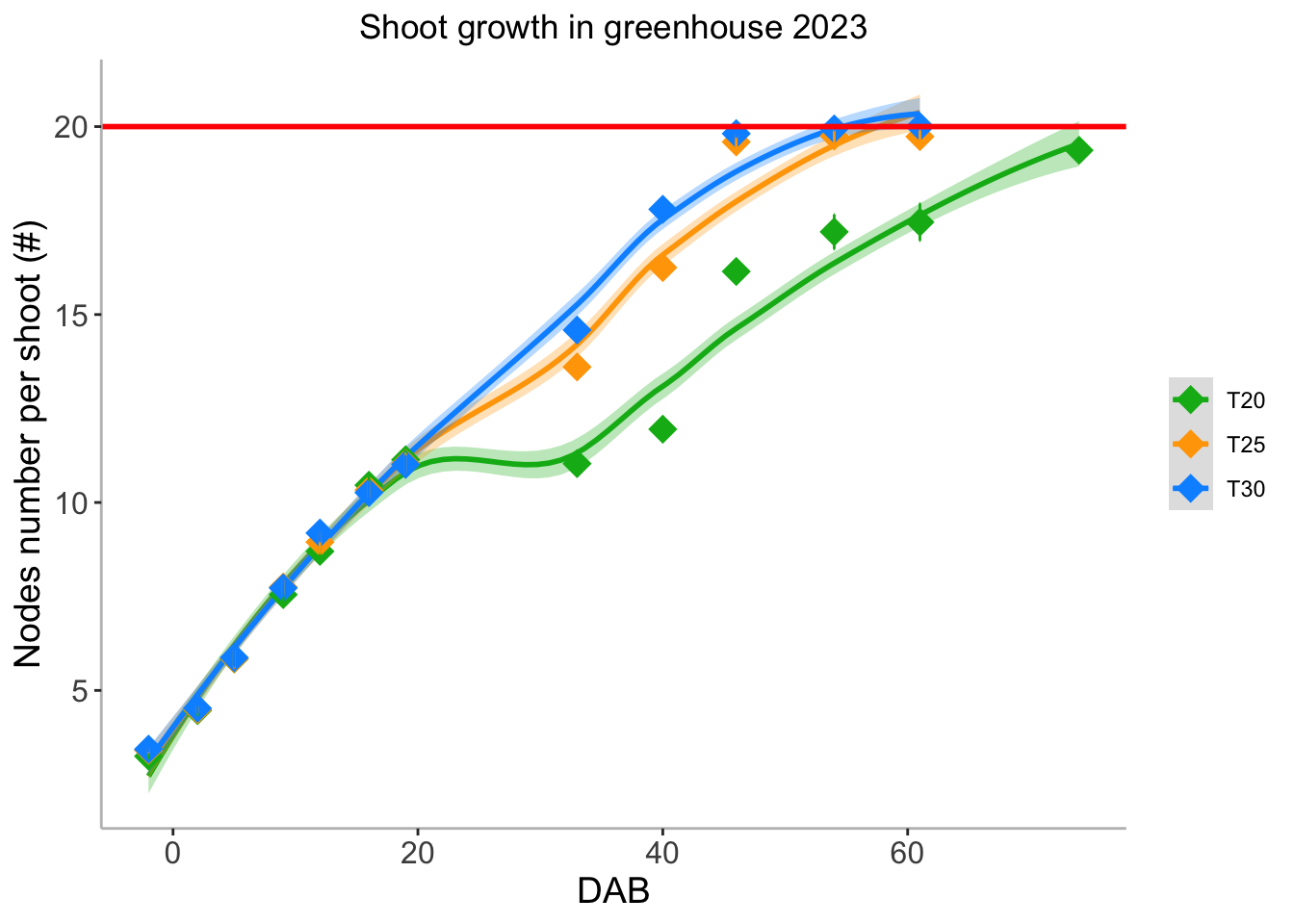


**Figure S9** – Shoot growth of Pinot Noir grapevines in the greenhouse for the year 2023, represented as the average number of nodes per shoot over days after budbreak (DAB). Different colors represent different greenhouse chambers (Green = T20, Orange = T25, Blue = T30). Markers represent the means ± confidence intervals. The red dotted line indicates the reference for the beginning of the water treatment.

**5. Stomatal conductance in different greenhouse chambers**


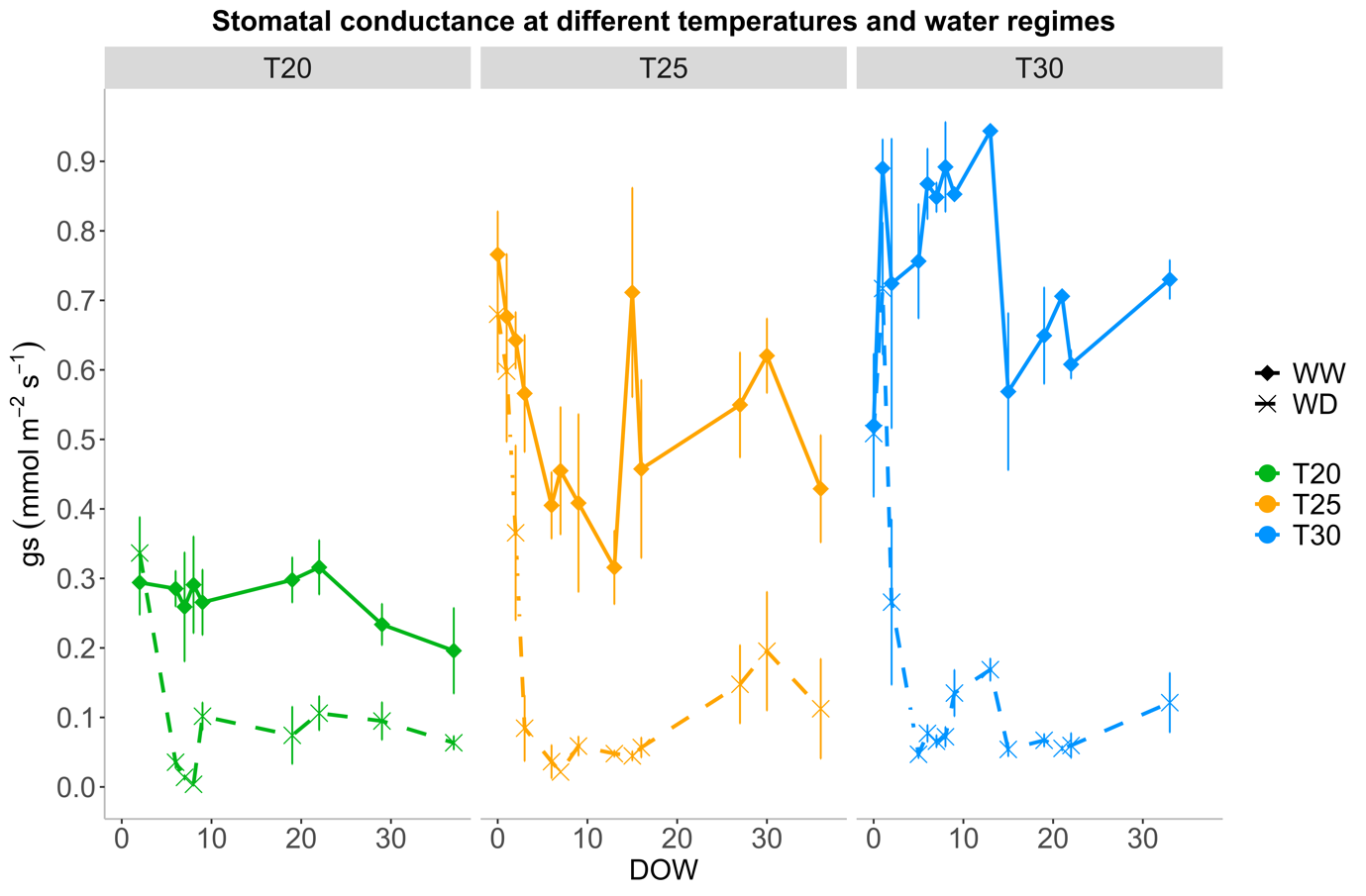


**Figure S10 –** Stomatal conductance (g_s_) over days of water deficit (DOW) of Pinot Noir grapevines grown in greenhouse chambers at 20°C (T20; green), 25°C (T25; orange) and 30°C (T30; blue) during the 2023. Plants were subjected to two water regimes: well-watered (WW; solid line with dots) and water deficit (WD; dashed line with crosses). Each value is the mean value at any given day of measurement, and vertical bars represent the standard error (n = 3–8). Significant differences (p-value<0.05) between WW and WD were observed after DOW>2.

**6. Statistical analysis**

**Table S3** – Linear model and analysis of variance (ANOVA) results used to test changes in osmotic potential at full turgor (π_100_) over time (days after budbreak; DAB) from Pinot noir leaves sampled in the semi-controlled field trial in 2023.

**lm(formula =** π_100_ **~ DAB, data = Semicontrolled field trial)**

Coefficients:

Estimate. Std. Error t value Pr(>|t|)

(Intercept) -0.9185249 0.0556368 -16.509 <2e-16 ***

DAB -0.0042168 0.0004904 -8.599 2e-12 ***

Residual standard error: 0.09517 on 67 degrees of freedom

Multiple R-squared: 0.5247, Adjusted R-squared: 0.5176

F-statistic: 73.95 on 1 and 67 DF, p-value: 2.003e-12

---

**Analysis of Variance Table**

Response: π_100_

Df Sum Sq Mean Sq F value Pr(>F)

DAB 1 0.66976 0.66976 73.95 2.003e-12 ***

Residuals 67 0.60682 0.00906

---

Signif. codes: 0 '***' 0.001 '**' 0.01 '*' 0.05 '.' 0.1 ' ' 1

**Table S4** – Linear model and analysis of variance (ANOVA) results used to test changes in osmotic potential at full turgor (π_100_) over time (days after budbreak; DAB) and temperature from Pinot noir leaves sampled in the 2022 greenhouse experiment.

**lm(formula =** π_100_ **~ DAB * Temp, data = Greenhouse 2022)**

Coefficients:

Estimate Std. Error t value Pr(>|t|)

(Intercept) -0.5786303 0.0665772 -8.691 6.24e-10 ***

DAB -0.0057194 0.0007359 -7.772 7.30e-09 ***

T25 -0.1065982 0.0989761 -1.077 0.290

DAB:T25 0.0011014 0.0010740 1.026 0.313

Residual standard error: 0.1082 on 32 degrees of freedom

Multiple R-squared: 0.7499, Adjusted R-squared: 0.7265

F-statistic: 31.98 on 3 and 32 DF, p-value: 9.463e-10

---

**Analysis of Variance Table**

Response: π_100_

Df Sum Sq Mean Sq F value Pr(>F)

DAB 1 1.10917 1.10917 94.7846 4.348e-11 ***

Temp 1 0.00131 0.00131 0.1122 0.7399

DAB:Temp 1 0.01231 0.01231 1.0517 0.3128

Residuals 32 0.37446 0.01170

---

Signif. codes: 0 ‘***’ 0.001 ‘**’ 0.01 ‘*’ 0.05 ‘.’ 0.1 ‘ ’ 1

**Table S5** – Linear model and analysis of variance (ANOVA) results used to test changes in osmotic potential at full turgor (π_100_) over time (days after budbreak; DAB), temperature (Temp) and their interaction (DAB x Temp) during the 2023 greenhouse season.

**lm(formula = π_100_ ~ DAB * Temp, data = Greenhouse 2023)**

Coefficients:

Estimate Std. Error t value Pr(>|t|)

(Intercept) -0.8526477 0.0642949 -13.262 < 2e-16 ***

DAB -0.0032553 0.0009606 -3.389 0.00116 **

T25 0.1339443 0.0892564 1.501 0.13800

T30 0.1985228 0.0892564 2.224 0.02941 *

DAB:T25 -0.0013897 0.0013534 -1.027 0.30806

DAB:T30 -0.0009490 0.0013534 -0.701 0.48553

Residual standard error: 0.1478 on 69 degrees of freedom

Multiple R-squared: 0.4938, Adjusted R-squared: 0.4571

F-statistic: 13.46 on 5 and 69 DF, p-value: 3.671e-09

---

**Analysis of Variance Table**

Response: π_100_

Df Sum Sq Mean Sq F value Pr(>F)

DAB 1 1.18811 1.18811 54.3830 2.776e-10 ***

Temp 2 0.25830 0.12915 5.9116 0.004269 **

DAB:Temp 2 0.02403 0.01201 0.5499 0.579519

Residuals 69 1.50745 0.02185

---

Signif. codes: 0 ‘***’ 0.001 ‘**’ 0.01 ‘*’ 0.05 ‘.’ 0.1 ‘ ’ 1

---

emmeans(lm, pairwise ~ Temp, adjust = "tukey")

contrast estimate SE df t.ratio p.value

T20 - T25 -0.0536 0.0423 69 -1.267 0.4186

T20 - T30 -0.1437 0.0423 69 -3.395 0.0032

T25 - T30 -0.0901 0.0410 69 -2.197 0.0789

**Table S6** – Linear model and analysis of variance (ANOVA) results used to test changes in osmotic potential at full turgor (π_100_) over days of water deficit (DOW), temperature (Temp), water deficit (WD) and the interaction DOW x WD x Temp during the 2023 greenhouse season.

**lm(formula = π_100_** **~ DOW * Water * Temp, data = Greenhouse 2023)**

Coefficients:

Estimate Std. Error t value Pr(>|t|)

(Intercept) -0.9739596 0.0341519 -28.518 < 2e-16 ***

DOW -0.0084186 0.0013355 -6.304 6.96e-09 ***

WW 0.0547218 0.0500940 1.092 0.277165

T25 -0.0032274 0.0469739 -0.069 0.945354

T30 0.1721924 0.0466096 3.694 0.000352 ***

DOW:WW -0.0001785 0.0018813 -0.095 0.924579

DOW:T25 0.0007057 0.0020388 0.346 0.729931

DOW:T30 0.0005588 0.0020957 0.267 0.790251

WW:T25 0.0475123 0.0687218 0.691 0.490858

WW:T30 0.0262187 0.0672429 0.390 0.697393

DOW:WW:T25 -0.0019295 0.0029052 -0.664 0.508043

DOW:WW:T30 0.0004293 0.0029590 0.145 0.884913

Residual standard error: 0.09582 on 105 degrees of freedom

Multiple R-squared: 0.7753, Adjusted R-squared: 0.7517

F-statistic: 32.93 on 11 and 105 DF, p-value: < 2.2e-16

---

**Analysis of Variance Table**

Response: π_100_

Df Sum Sq Mean Sq F value Pr(>F)

DOW 1 2.25531 2.25531 245.6116 < 2.2e-16 ***

Water 1 0.14870 0.14870 16.1942 0.0001081 ***

Temp 2 0.90371 0.45185 49.2086 8.358e-16 ***

DOW:Water 1 0.00427 0.00427 0.4648 0.4968852

DOW:Temp 2 0.00418 0.00209 0.2275 0.7968769

Water:Temp 2 0.00404 0.00202 0.2201 0.8028252

DOW:Water:Temp 2 0.00599 0.00299 0.3260 0.7225251

Residuals 105 0.96415 0.00918

---

emmeans(lm, pairwise ~ Temp, adjust = "tukey")

contrast estimate SE df t.ratio p.value

T20 - T25 -0.016 0.0222 105 -0.721 0.7515

T20 - T30 -0.199 0.0220 105 -9.053 <.0001

T25 - T30 -0.183 0.0229 105 -7.993 <.0001

Results are averaged over the levels of: Water

**Table S7** – Partial least squares (PLS) analysis and loadings showing the impact of the different osmolytes concentration on the osmotic potential at full turgor (π_100_) across all greenhouse trials and the semi-controlled field trial.

Data: X dimension: 277 7

Y dimension: 277 1

Fit method: kernelpls

Number of components considered: 7

VALIDATION: RMSEP

Cross-validated using 10 random segments.

(Intercept) 1 comps 2 comps 3 comps 4 comps 5 comps 6 comps 7 comps

CV 0.248 0.09878 0.09467 0.09082 0.08821 0.08686 0.08850 0.08760

adjCV 0.248 0.09875 0.09457 0.09070 0.08808 0.08670 0.08825 0.08736

TRAINING: % variance explained

1 comps 2 comps 3 comps 4 comps 5 comps 6 comps 7 comps

X 47.26 65.70 79.00 87.01 89.45 97.16 100.00

OSM_P100_MPa 84.29 86.03 87.23 88.04 88.53 88.59 88.67

(Intercept) 1 comps

R2 -0.00726 0.84017

MSEP CV / adjCV 0.06192 0.009703

Loadings

K_gl -0.44349556

Mg_gl -0.04197775

Ca_gl -0.42691731

Glucose_gl -0.48540535

Fructose_gl -0.44443719

Sucrose_gl -0.39634355

Myo-Inositol_gl -0.18594876

**Table S11** – Linear model and analysis of variance (ANOVA) results used to test changes in osmotic potential at full turgor (π_100_) over time (days after budbreak; DAB), experimental site (SITE), water treatment (Water) and their interaction (DAB x Temp x Water) across all the experimental sites and season.

**lm(formula =** π_100_ **~ DAB * Water * SITE, data = Osmolytes)**

Coefficients:

Estimate Std. Error t value Pr(>|t|)

(Intercept) -0.7239347 0.0391940 -18.471 <2e-16 ***

DAB -0.0045792 0.0004455 -10.279 <2e-16 ***

WaterWD 0.0556457 0.1279946 0.435 0.6640

SITET25 0.0479759 0.0552069 0.869 0.3855

SITET30 0.1153441 0.0605140 1.906 0.0575 .

SITEsemicontr -0.1945902 0.0810523 -2.401 0.0169 *

DAB:WaterWD -0.0003141 0.0012763 -0.246 0.8058

DAB:SITET25 -0.0003422 0.0006502 -0.526 0.5991

DAB:SITET30 0.0004683 0.0007715 0.607 0.5442

DAB:SITEsemicontr 0.0003623 0.0007677 0.472 0.6373

WaterWD:SITET25 -0.0352216 0.1685863 -0.209 0.8346

WaterWD:SITET30 0.2149050 0.2041373 1.053 0.2933

WaterWD:SITEsemicontr NA NA NA NA

DAB: WaterWD:SITET25 -0.0001145 0.0017401 -0.066 0.9476

DAB: WaterWD:SITET30 -0.0034348 0.0024918 -1.378 0.1690

DAB: WaterWD:SITEsemicontr NA NA NA NA

Residual standard error: 0.1214 on 317 degrees of freedom

Multiple R-squared: 0.7515, Adjusted R-squared: 0.7413

F-statistic: 73.73 on 13 and 317 DF, p-value: < 2.2e-16

---

**Analysis of Variance Table**

Response: π_100_

Df Sum Sq Mean Sq F value Pr(>F)

DAB 1 11.5267 11.5267 782.6997 < 2.2e-16 ***

Water 1 0.2131 0.2131 14.4687 0.000171 ***

SITE 3 2.2854 0.7618 51.7283 < 2.2e-16 ***

DAB: Water 1 0.0112 0.0112 0.7624 0.383245

DAB:SITE 3 0.0212 0.0071 0.4802 0.696300

Water:SITE 2 0.0267 0.0134 0.9072 0.404698

DAB:Water:SITE 2 0.0315 0.0157 1.0691 0.344556

Residuals 317 4.6684 0.0147

---

Signif. codes: 0 ‘***’ 0.001 ‘**’ 0.01 ‘*’ 0.05 ‘.’ 0.1 ‘ ’ 1

**7. Osmolytes contributions and concentrations**

**Table S8** – Average calculated contribution (%) of individual osmolytes, osmolyte categories, and total contribution of the measured osmolytes to the osmotic potential at full turgor (π_100_) across all experiments, calculated as the mean of all sites included in the analysis. Theoretical osmotic potential from each osmolyte was calculated from the concentrations measured in the leaf sap sample with HPIC and compared against the total osmotic potential measured with the pressure-vapor osmometer.

| Osmolytes | % |
| --- | --- |
| K | 3.8 |
| Mg | 5.1 |
| Ca | 5.5 |
| Myo-Inositol | 2.6 |
| Glucose | 17.1 |
| Fructose | 17.9 |
| Sucrose | 2.5 |
| Total cations | 14.4 |
| Total sugars | 40.1 |
| Total | 54.4 |

**Table S9** – Average calculated contribution (%) of individual osmolytes, osmolyte categories, and total contribution of the measured osmolytes to the osmotic potential at full turgor (π_100_) at different days after budbreak (DAB) in the semi-controlled field trial sampling season in 2023. Theoretical osmotic potential from each osmolyte was calculated from the concentrations measured in the leaf sap sample with HPIC and compared against the total osmotic potential measured with the pressure-vapor osmometer.

|  | DAB 75 | DAB 98 | DAB 112 | DAB 133 | DAB 145 |
| --- | --- | --- | --- | --- | --- |
| K | 3.7 | 4.5 | 4.8 | 6.3 | 6.4 |
| Mg | 3.9 | 4.1 | 4.3 | 4.3 | 4.3 |
| Ca | 7.6 | 7.5 | 8.0 | 9.7 | 9.2 |
| Myo-Inositol | 2.8 | 2.5 | 1.9 | 1.7 | 1.7 |
| Glucose | 20.2 | 18.9 | 18.3 | 16.7 | 20.6 |
| Fructose | 16.9 | 15.6 | 18.2 | 17.2 | 21.7 |
| Sucrose | 3.2 | 3.2 | 2.6 | 2.4 | 2.5 |
| Total cations | 15.2 | 16.1 | 17.2 | 20.4 | 19.9 |
| Total sugars | 43.2 | 40.2 | 40.9 | 38.1 | 46.3 |
| Total | 58.4 | 56.3 | 58.0 | 58.5 | 66.3 |

**Table S10** – Average calculated contribution (%) of individual osmolytes, osmolyte categories, and total contribution of the measured osmolytes to the osmotic potential at full turgor (π_100_) at different days after budbreak (DAB) during the 2023 greenhouse season trial. Theoretical osmotic potential from each osmolyte was calculated from the concentrations measured in the leaf sap sample with HPIC and compared against the total osmotic potential measured with the pressure-vapor osmometer.

| DAB | 3 | 24 | 56 | 86 | 3 | 24 | 56 | 86 | 3 | 24 | 56 | 86 |
| --- | --- | --- | --- | --- | --- | --- | --- | --- | --- | --- | --- | --- |
| Temp | T20 | T20 | T20 | T20 | T25 | T25 | T25 | T25 | T30 | T30 | T30 | T30 |
| K | 2.6 | 3.3 | 4.2 | 3.3 | 3.1 | 3.2 | 4.3 | 4.0 | 2.5 | 3.1 | 4.3 | 4.8 |
| Mg | 6.7 | 7.7 | 6.3 | 4.0 | 7.7 | 9.8 | 8.0 | 6.2 | 7.8 | 10.8 | 7.8 | 6.8 |
| Ca | 4.8 | 7.5 | 5.4 | 4.5 | 5.1 | 6.9 | 6.8 | 5.9 | 3.3 | 6.8 | 7.0 | 6.9 |
| Myo-Inositol | 2.9 | 2.8 | 2.0 | 1.3 | 2.6 | 2.1 | 1.9 | 2.3 | 2.5 | 1.8 | 1.9 | 2.6 |
| Glucose | 21.4 | 14.4 | 15.6 | 26.4 | 14.6 | 13.0 | 16.2 | 16.5 | 17.2 | 13.7 | 16.2 | 20.1 |
| Fructose | 24.9 | 18.0 | 14.4 | 28.9 | 17.3 | 16.1 | 15.7 | 14.7 | 20.5 | 16.9 | 16.0 | 22.3 |
| Sucrose | 1.6 | 1.2 | 1.7 | 1.2 | 2.1 | 1.2 | 1.0 | 3.0 | 1.5 | 1.2 | 1.2 | 3.5 |
| Cations | 14.1 | 18.5 | 16.0 | 11.8 | 16.0 | 19.9 | 19.1 | 16.0 | 13.5 | 20.7 | 19.0 | 18.5 |
| Sugars | 50.8 | 36.4 | 33.7 | 57.8 | 36.6 | 32.5 | 34.7 | 36.4 | 41.6 | 33.6 | 35.2 | 48.4 |
| Total osmolites | 64.9 | 54.9 | 49.7 | 69.6 | 52.5 | 52.4 | 53.8 | 52.5 | 55.2 | 54.3 | 54.2 | 66.9 |

**
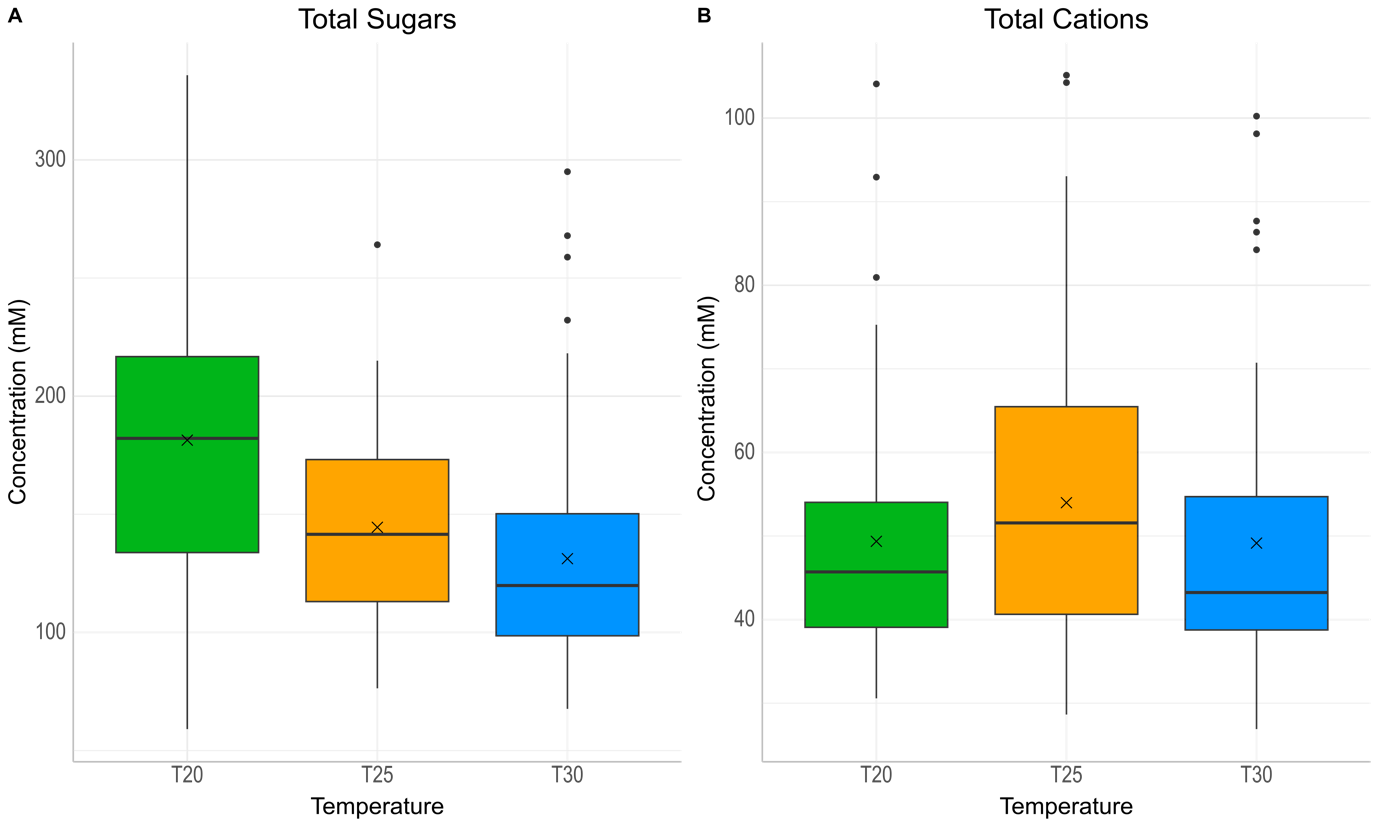
Figure S11 –** Boxplot showing the total concentration of sugars (A) and cations (B) in millimolar (mM) in Pinot Noir grapevines grown in greenhouse chambers at 20°C (T20; green), 25°C (T25; orange), and 30°C (T30; blue) during the 2023 season. The cross mark represents the average concentration within each treatment group.
